# The unique gut microbiome of giant pandas involved in protein metabolism contributes to the host’s dietary adaption to bamboo

**DOI:** 10.1186/s40168-023-01603-0

**Published:** 2023-08-14

**Authors:** Feilong Deng, Chengdong Wang, Desheng Li, Yunjuan Peng, Linhua Deng, Yunxiang Zhao, Zhihao Zhang, Ming Wei, Kai Wu, Jiangchao Zhao, Ying Li

**Affiliations:** 1 Guangdong Provincial Key Laboratory of Animal Molecular Design and Precise Breeding, College of Life Science and Engineering, Foshan University, Foshan, China; 2 School of Life Science and Engineering, Foshan University, Guangdong, China; 3 China Conservation and Research Center of Giant Panda, Key Laboratory of SFGA on Conservation Biology of Rare Animals in The Giant Panda National Park (CCRCGP), Sichuan 611830 Dujiangyan, China; 4 Department of Animal Science, Division of Agriculture, University of Arkansas, AR Fayetteville, USA

## Abstract

**Background:**

The gut microbiota of the giant panda (*Ailuropoda melanoleuca*), a global symbol of conservation, are believed to be involved in the host’s dietary switch to a fibrous bamboo diet. However, their exact roles are still largely unknown.

**Results:**

In this study, we first comprehensively analyzed a large number of gut metagenomes giant pandas (*n* = 322), including 98 pandas sequenced in this study with deep sequencing (Illumina) and third-generation sequencing (nanopore). We reconstructed 408 metagenome-assembled genomes (MAGs), and 148 of which (36.27%) were near complete. The most abundant MAG was classified as *Streptococcus alactolyticus*. A pairwise comparison of the metagenomes and meta-transcriptomes in 14 feces revealed genes involved in carbohydrate metabolism were lower, but those involved in protein metabolism were greater in abundance and expression in giant pandas compared to those in herbivores and omnivores. Of note, *S. alactolyticus* was positively correlated to the KEGG modules of essential amino-acid biosynthesis. After being isolated from pandas and gavaged to mice, *S. alactolyticus* significantly increased the relative abundance of essential amino acids in mice jejunum.

**Conclusions:**

The study highlights the unique protein metabolic profiles in the giant panda’s gut microbiome. The findings suggest that *S. alactolyticus* is an important player in the gut microbiota that contributes to the giant panda’s dietary adaptation by more involvement in protein rather than carbohydrate metabolism.

Video Abstract

**Supplementary Information:**

The online version contains supplementary material available at 10.1186/s40168-023-01603-0.

## Introduction

The giant panda (*Ailuropoda melanoleuca*), a flagship species for wildlife conservation worldwide, was upgraded from an endangered species to a vulnerable category in 2016 (The IUCN Red List of Threatened Species, 2016) [1, 2]. Giant panda belongs to the family Ursidae, which includes both carnivorous and omnivorous members [3]. The giant panda’s diet consists exclusively of highly fibrous bamboo, despite its typical carnivore-like gastrointestinal tract, which is intriguing to researchers and conservationists [4, 5].

Since the genome of the giant panda lacks the enzymes required for digesting cellulose and hemicellulose [6], scientists have focused on the roles of gut microbes in the giant pandas’ health and nutrition. A pioneering study reported the presence of putative cellulose genes in the gut microbiota of giant pandas [7]. However, the composition and functional potential of the gut microbiota in giant pandas were more similar to those of other carnivores than herbivores [8, 9], suggesting low-fiber digestion in giant pandas. Our previous studies have also shown that captivity [10] and reintroduction [11] significantly altered the gut microbiota of giant pandas. Furthermore, certain gut microbiota of giant pandas can potentially detoxify cyanide compounds [12]. Another interesting finding is the similarity in macronutrient energy ratios of the pandas’ diets with those of carnivores [13].

Despite the growing body of knowledge about the giant panda’s microbial composition and structures, much still remains unknown regarding their functions. For example, metagenome-assembled genomes (MAGs) are critical to better understanding metagenome datasets and guiding functional analyses. MAGs have been constructed in many mammalian species, such as humans [14], mice [15], and livestock, including cows [16, 17], goats [18], pigs [19, 20], buffalo [21], and chickens [22, 23]. Although a limited number of studies have investigated the MAGs of giant pandas [24], the sample sizes of the study were small, and the sequencing depth was shallow. A comprehensive reconstruction of MAGs in giant pandas is still lacking. In addition, although metagenome studies provide insight into the microbial functional potential in giant pandas, they do not reveal gene expression profiles; therefore, genes and metabolic pathways actively involved in nutrient digestion are still largely unknown.

To fill these knowledge gaps, this study aimed to (1) reconstruct high-quality MAGs for better understanding of microbiome functions by deep sequencing of a large number of fecal samples from giant pandas; (2) examine the contribution of gut microbiota to panda’s dietary changes by detecting genes highly expressed in nutrient metabolism with a pairwise comparison of metagenomic and meta-transcriptomic data.

## Results

### Metagenomic assembled genomes (MAGs) in giant pandas

**Fig. 1:**
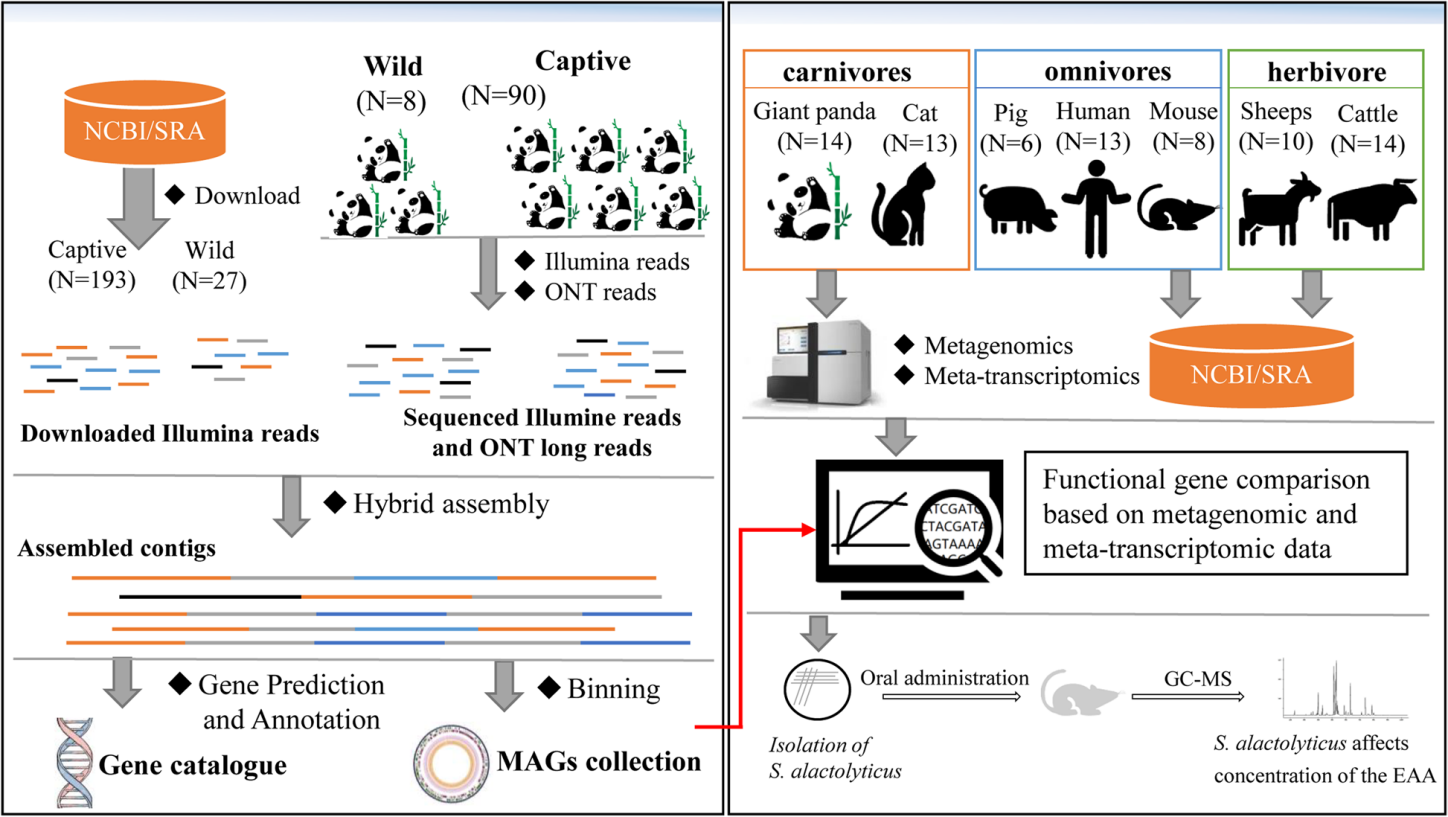
Schematic overview of the experimental design

To construct high-quality MAGs, we first performed deep sequencing of fecal samples collected from 98 giant pandas with a combination of the next- (i.e., Illumina Hi-Seq) and third-generation (i.e., Oxford Nanopore Technologies, ONT) sequencing (Fig. 1). Next-generation sequencing of fecal samples from 90 captive giant pandas yielded an average of 27.42 Gbp per sample, ranging from 20.08 to 33.48 Gbp. In addition, three pooled samples were sequenced on the ONT platform, which resulted in an average of 5,460,747 nanopore long reads per sample (66.58 Gbp/sample) (Table S1). Of note, higher sequencing depths were achieved for the eight samples from wild giant pandas; the average sequencing depth of Illumina reads for each sample exceeded 31 Gbp (ranging from 28.97 to 35.62 Gbp). Two pooled libraries were generated and sequenced for samples collected from the wild giant pandas using ONT, which generated 6,328,671 nanopore reads per sample (63.48 Gbp/sample).

**Fig. 2:**
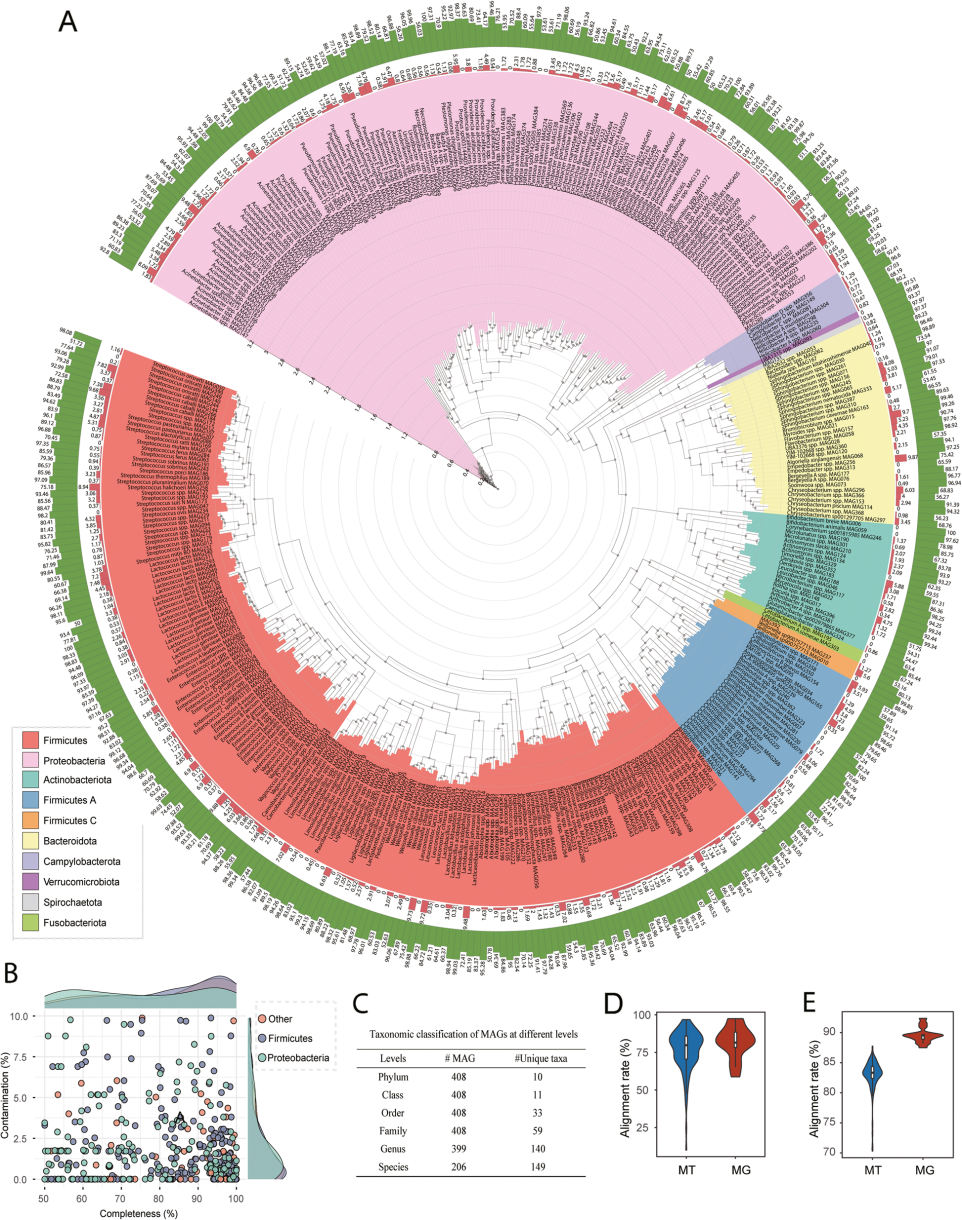
Taxonomic annotation and phylogenetic tree of 408 metagenome-assembled genomes (MAGs). **A** The outermost circle (green) and outer cycle (orange) represent completeness and contamination of MAGs. The different colors of the background of clades represent different bacterial phyla. The tree was constructed using PhyloPhlan (v3.0.2) and visualized using Interactive Tree of Life (iTOL, v6.5.2). **B** Distribution of completeness and contamination of these MAGs. **C** Taxonomic classification of MAGs at different levels. **D** Alignment rate to the MAGs for metagenomic clean reads (MG, *N* = 125) and meta-transcriptomic clean reads (MT, *N* = 14). **E** Alignment rate to the gene collection of giant pandas in the gut for metagenomic clean reads (MG, *N* = 125) and meta-transcriptomic clean reads (MT, *N* = 14)

To expand the width and depth of our data, we downloaded two published datasets to include in our MAG construction, one from Guo et al. [25, 26] (*n* = 198) with an average of 18.93 Gbp/sample totaling 3.72 Tb, and the other from Wu and colleagues [27] (*n* = 27) with an average of 6.78 Gbp/sample, resulting in a total of 322 metagenome. These metagenomes and five pooled ONT data of giant pandas were included to construct MAGs using a hybrid assembly strategy [28]. We first assembled 610 MAGs with completeness ≥ 50%, contamination ≤ 10%, and length ≥ 200 kb. These MAGs were further dereplicated at 99% average nucleotide sequence identity (ANI) to exclude repeated MAGs. Ultimately, we generated a non-redundant gut microbial genome draft set of giant pandas comprised of 408 MAGs (Fig. 2A). Among these MAGs, 148 (36.27%) were near complete (≥ 90% completeness) with low contamination (≤ 5%) and were considered high-quality MAGs [29] (Fig. 2B). The genome size of 408 MAGs ranged from 1.07 to 6.08 Mb, with an average size of 2.62 Mb, and the N50 size of MAGs ranged from 3875 to 368,647 bp (Table S2). Among the high-quality MAGs, 31 MAGs have only one scaffold with an average genome length of 2.56 Mb (ranging from 1.49 to 5.74 Mb), showing great contiguity of the genome assembly.

We identified 80 MAGs that meet the standards of the Genomic Standards Consortium (GSC) for high-quality genome: MAG with 23S, 16S, and 5S rRNA genes and at least 18 tRNAs together with completeness ≥ 90% and contamination ≤ 5% [30] (Table S2).

To determine the quality of our MAGs by calculating the coverage of microbial genomes for different giant panda populations, we mapped metagenomic clean reads (rRNA filtered) to our 408 MAGs. The average alignment rate for metagenomic reads was 76.47% (Fig. 2D).

Taxonomy of the MAGs was subsequently assigned using the Genome Taxonomy Database Toolkit (GTDB-Tk “classify_wf”). The 408 MAGs were classified into 59 bacterial families belonging to 10 phyla (Fig. 2C). The majority of the MAGs were assigned to either Firmicutes (204 MAGs, 50.00%) or Proteobacteria (139 MAGs, 34.07%), followed by Bacteroidota (33 MAGs, 8.09%) and Actinobacteriota (21 MAGs, 5.15%). At the genus level, the most abundant five genus were *Streptococcus* (31.36%, average relative abundance), *Clostridium* (24.13%), *Escherichia* (19.09%), *Leuconostoc* (5.48%), and *Pseudomonas E* (4.18%) (Figure S1). At the MAGs level, *Streptococcus alactolyticus *MAG086 was the most abundant MAG with an average relative abundance of 35.08%, followed by *Clostridium *spp. MAG077 (11.91%), *Escherichia flexneri *MAG203 (7.41%), and *E. flexneri *MAG108 (7.41%). The genome annotations of the top 15 MAGs are shown in Figure S2. Of note, several MAGs contain genes involved in cellulose and hemicellulose degradation, including endo-1,4-beta-xylanase (EC 3.2.1.8), cellobiose phosphorylase (EC 2.4.1.20), and endocellulase (EC 3.2.1.4). Most of these genes were observed in three MAGs: MAG182, MAG052, and MAG085, belonging to the *Paenibacillus* genus, although species-level classification remains undetermined. Furthermore, MAGs from *Escherichia flexneri* (MAG108 and MAG203) and *Escherichia coli* (MAG390) hosted at least one gene associated with cellulose and hemicellulose degradation (Table S2).

### Meta-transcriptomic analysis of the gut microbiota in giant pandas

Although several studies have analyzed the metagenomics of giant pandas, which provide insight into the functional potentials of these microorganisms, little is known about the genes that are expressed from the active bacteria. Therefore, after a comprehensive analysis of the MAGs of the gut microbiota in giant pandas, we next investigated the gene expression profiles of the active bacteria in the giant pandas by meta-transcriptomic sequencing of 14 fecal samples from captive giant pandas. Of note, these 14 samples were also included in the metagenome sequencing described above, allowing us to make a pairwise comparison of the meta-transcriptomes and metagenomes in the same animals.

After removing low-quality reads, host contamination, and contaminant rRNA reads, an average of 9.83 Gbp high-quality data per sample ranging from 7.84 to 12.58 Gbp were obtained for downstream analysis (Table S3). The alignment rate of the 14 meta-transcriptome data to the gene catalog was even higher than the metagenomic data, with a mean of 89.59% across the 14 meta-transcriptomic samples ranging from 87.50 to 92.38% (Fig. 2E).

At the species level, the dominant species revealed by metagenomics was *E. flexneri*, with an average relative abundance of 27.10%, followed by *S. alactolyticus* (24.34%) and *E. coli* (6.08%). By contrast, the most abundant species detected by meta-transcriptomic data was *S. alactolyticus*, with a mean abundance of 45.45%, followed by *E. flexneri* (10.49%) and *Leuconostoc lactis A* (3.66%). Of note, although *E. coli* was the third abundant species based on metagenome, it was only ranked sixth with an average abundance of 2.38% according to the meta-transcriptomic data (Figure S3).

### Microbial metabolic pathways involved in nutrient metabolism in pandas compared to other animals

**Fig. 3:**
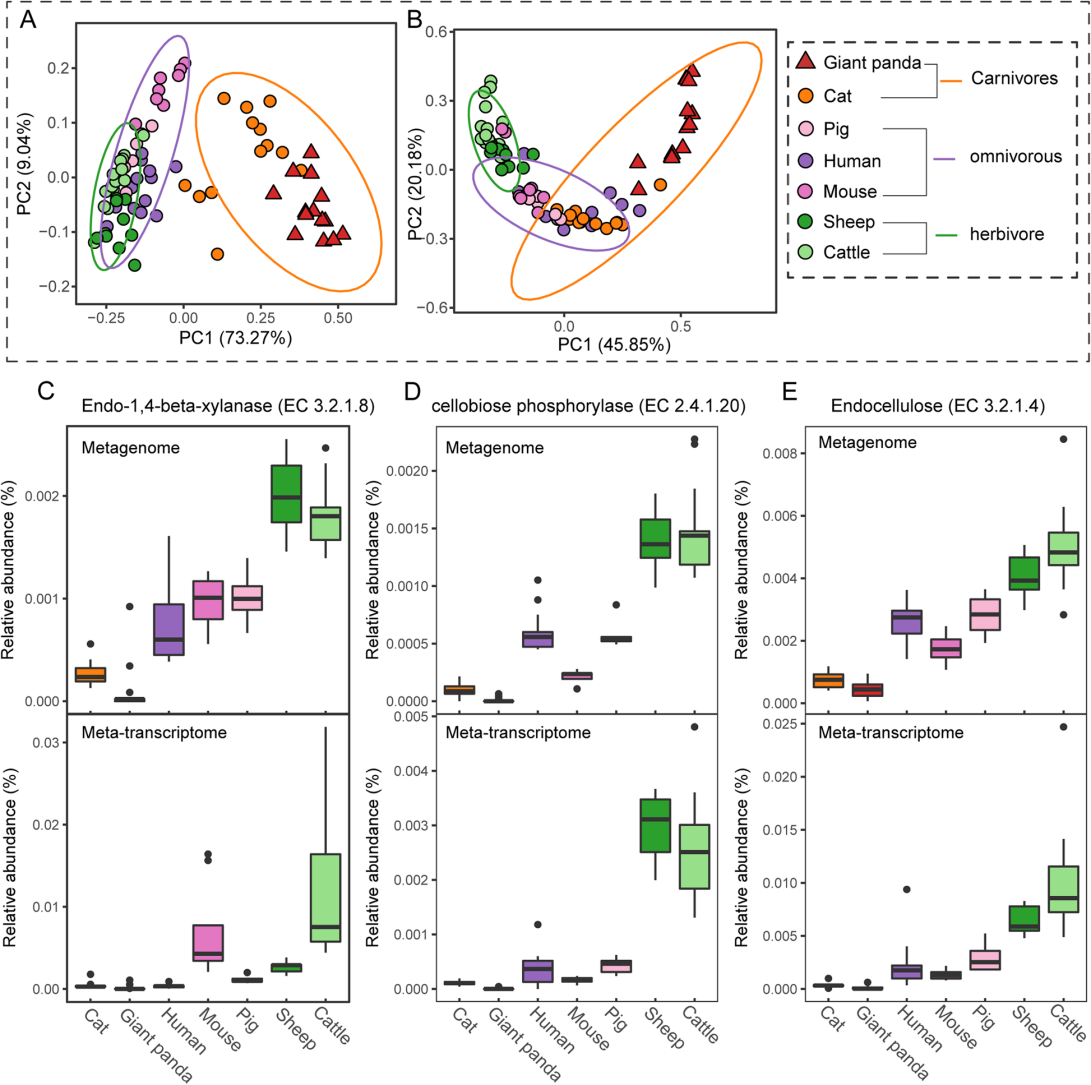
Composition and expression pattern of dietary fiber metabolic enzyme-related genes in the gut microbiota of different host species. Bray–Curtis diversity plot based on dietary fiber metabolic enzyme-related gene families. Bray–Curtis distance was measured by metagenomic abundance (**A**) and meta-transcriptomic expression (**B**) of these gene families. The ellipses were calculated and drawn with a 0.95 confidence level. Metagenomic abundance and meta-transcriptomic expression of crucial genes involved in hemicellulose degradation (EC 3.2.1.8, shown in **C**) and cellulase degradation (EC 3.2.1.4 and EC 3.2.1.20, shown in **D** and **E**) of different host species

Phylogenetically, giant pandas belong to the Carnivora group of mammals, but they thrive on a bamboo-dominated diet. To explore the microbial adaptation to, and functions in, this dietary shift in giant pandas, we investigated the microbial metabolic pathways in major nutrients (e.g., carbohydrate, amino acids) metabolism and compared them to mammals within three dietary patterns: herbivores (cattle and sheep), omnivores (pig, mouse, and human), and carnivores (cat) based on both metagenomic abundance and meta-transcriptomic expression. We first selected 27 gene families (Table S4) encoding enzymes involved in cellulose, hemicellulose, or lignose degradation to calculate beta diversity distance (Bray–Curtis) between different host species. Figure 3A shows the PCoA plot based on the metagenomic abundance of these selected gene families. The microbial gene families encoding metabolic pathways in plant cell wall degradation in pandas were more similar to those of cats. Interestingly, the membership and abundance of these genes in carnivores were significantly different from those in herbivores (ANOSIM, *R* = 0.81, *P* = 0.001) and omnivores (ANOSIM, *R* = 0.93, *P* = 0.001) (Fig. 3A). Bray–Curtis distance based on gene expression profiles of these gene families according to meta-transcriptomic data showed a similar pattern that carnivores were significantly separated from omnivores (ANOSIM, *R* = 0.37, *P* = 0.001) and herbivores (ANOSIM, *R* = 0.75, *P* = 0.001) (Fig. 3B).

Consistently, comparison of relative abundance and expression of key genes involved in cellulose- and hemicellulose degradation such as endo-1,4-beta-xylanase (EC 3.2.1.8), cellobiose phosphorylase (EC 2.4.1.20), and endo-cellulase (EC 3.2.1.4) revealed similar patterns among the three groups of mammals. Both abundance (metagenome data) and expression (meta-transcriptome data) of genes encoding endo-1,4-beta-xylanase (Fig. 3C), cellobiose phosphorylase (Fig. 3D), and endo-cellulase (Fig. 3E) in carnivores (cat and giant pandas) were significantly lower than those in cattle and sheep (*P* ≤ 0.01).

**Fig. 4:**
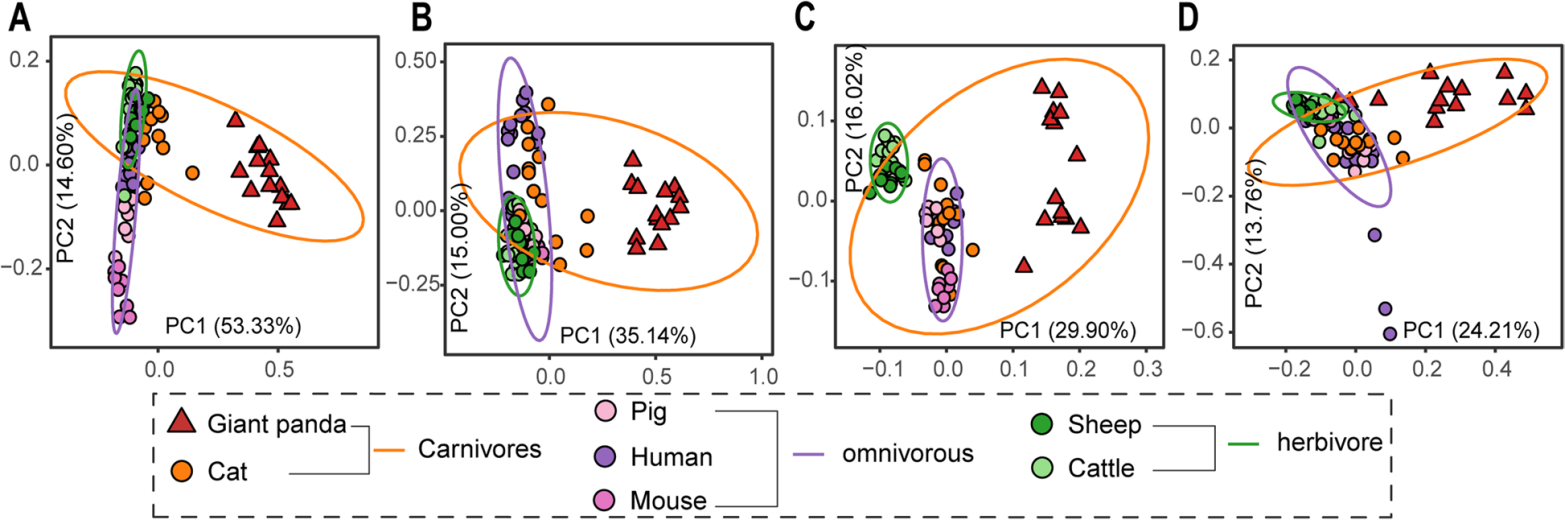
Abundance and expression pattern of genes involved in amino acid metabolism in gut microbiotas of different host species. Bray–Curtis distance between groups calculated with gene families encoding amino acid metabolic enzyme based on gene abundance from metagenomic (**A** AA degradation, **C** AA biosynthesis) and expression from meta-transcriptomic data (**B** AA degradation, **D** AA biosynthesis). The ellipses were calculated and drawn with a 0.95 confidence level

Bamboo protein is the primary energy source of giant pandas, according to previous studies [9, 13]. Therefore, we analyzed the abundance and expression of gene families involved in amino acid metabolism, including 61 gene families (Table S4) encoding enzymes for amino acid degradation and 98 gene families encoding enzymes for biosynthesis of amino acids in the gut or rumen microbiomes of giant pandas and other host species. Beta diversity (Bray–Curtis) based on gene abundance in the metagenomic data showed that different host species differed significantly in the genes involved in amino acid degradation (ANOSIM, *P* ≤ 0.05), except for the pairwise comparison between cats and pigs (*R* = 0.12, *P* = 0.137) (Fig. 4A). Regarding the gene expression profiles of these genes, all host species were distinct from one another (Fig. 4B), although cats and giant pandas were more similar in their gene expression patterns. As to dietary patterns, carnivores (giant panda and cat) were significantly distinguished from omnivores (*R* = 0.616, *P* = 0.001) and herbivores (*R* = 0.576, *P* = 0.001), while omnivores and herbivores showed statistical differences but with a lower *R* value (*R* = 0.203, *P* = 0.002). In terms of amino acid biosynthesis, the giant panda was significantly different from other host species (*P* < 0.01) on the PCoA plots based on both the metagenomic (Fig. 4C) and meta-transcriptomic (Fig. 4D) dataset.

### Essential amino acids biosynthesis by the gut microbiome of giant pandas

**Fig. 5:**
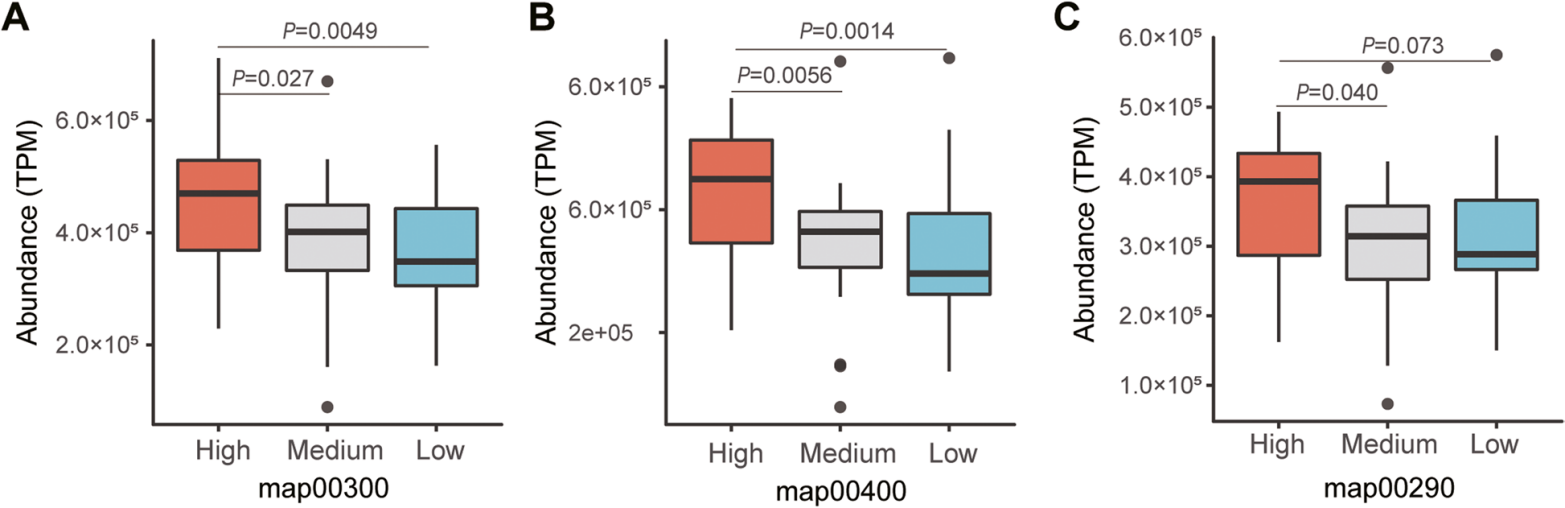
Relative abundance of essential-amino biosynthesis KEGG pathway in giant pandas with different *S. alactolyticus* levels. The high, medium, and low on the *x*-axis of plots **A**, **B**, and **C** represent the high, medium, and low abundance of *S. alactolyticus* in giant pandas samples, respectively. *Y*-axis is the normalized total abundance of the KEGG module related to essential amino acid biosynthesis. Statistical significance (*p* value) was calculated by unpaired two-tailed *t* test

**Fig. 6:**
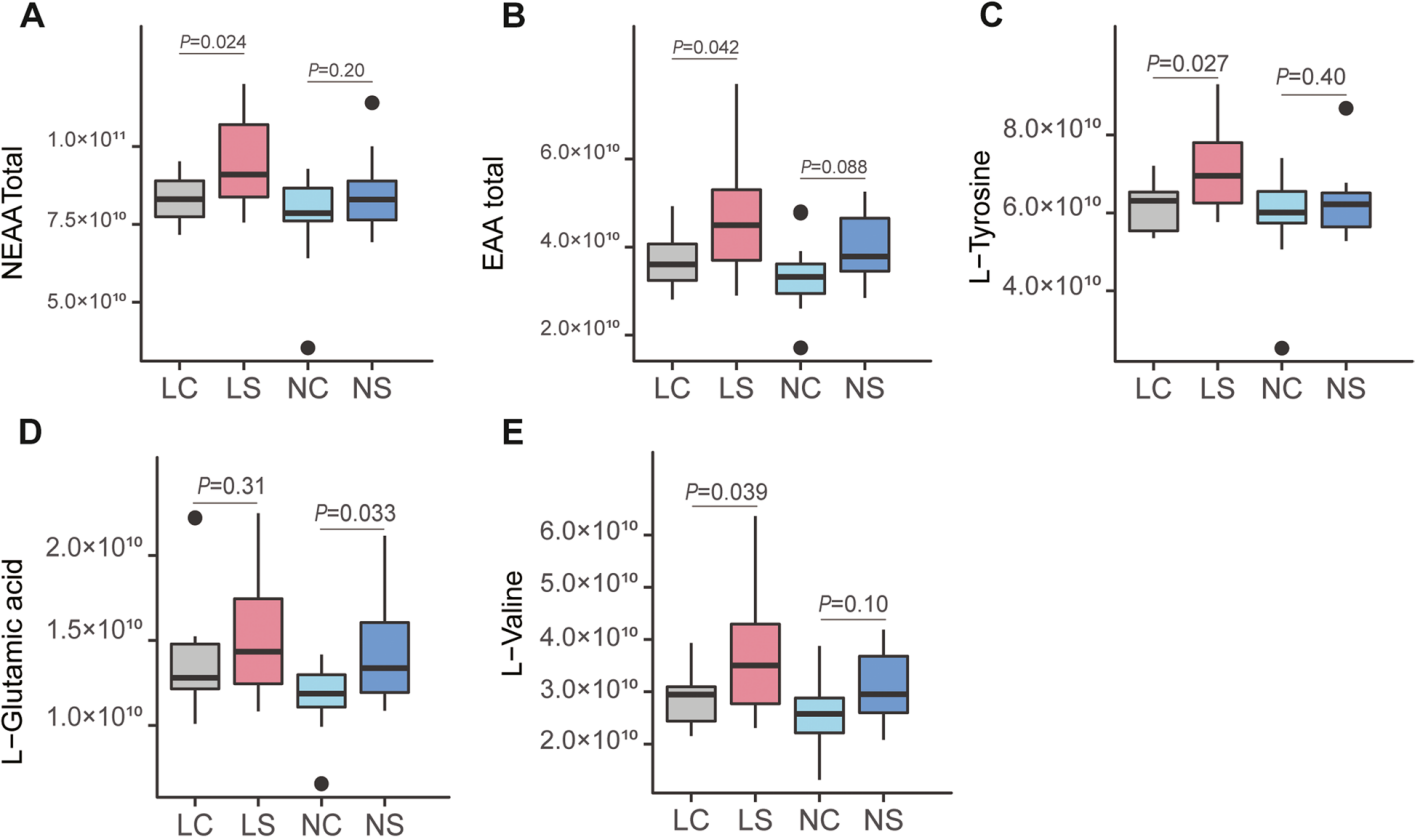
Comparison of amino acid abundance for the different treatment groups in mice. **A** and **B** indicated the comparison of the total abundance of the non-essential amino acids (**A**) and essential amino acids (**B**) for different treatment mice groups. Boxplots of **C**-**E** showed the abundance of L-tyrosine, L-glutamic acid, and L-valine in different mice groups, respectively. Statistical significance (*p* value) was calculated by unpaired two-tailed *t* test

Plant proteins often lack or are insufficient in some essential amino acids required by animals. To explore whether gut microbiome helped the host to synthesize essential amino acids, we analyzed the abundance of genes involved in essential amino-acid biosynthesis in *S. alactolyticus*, the most abundant bacterial taxa with the greatest gene expression levels in the gut of the giant pandas. We regrouped the captive giant pandas into three groups based on the relative abundance (RA) of *S. alactolyticus*: high [RA ≥ 50%, *n* = 28], medium (50% > RA ≥ 25%, *n* = 29), and low (RA < 25%, *n* = 33). The KEGG pathways related to essential amino-acid biosynthesis, including map00290 (Valine, leucine, and isoleucine biosynthesis), map00300 (Lysine biosynthesis), and map00400 (phenylalanine, tyrosine, and tryptophan biosynthesis), were analyzed based on metagenomic sequencing reads. Comparative analysis revealed that the abundance of map00300 KEGG pathway (Fig. 5A) and map00400 KEGG pathway (Fig. 5B) of the High group was significantly greater than that of the medium (map00300: *P* = 0.027, map00400: *P* = 0.0056) and low (map00300: *P* = 0.0049, map00400: *P* = 0.0014) groups. For the map00290 KEGG pathway (Fig. 5C), its abundance in the high group was significantly higher than that of the medium group (*P* = 0.040), while trended to be higher in the high group than in the low group with no statistical significance (*P* = 0.073) Fig. 6.

To verify its role in synthesizing the essential amino acid in the gut, we first isolated *S. alactolyticus* from pandas, then administered it to BALB/c mice with low and normal protein diets via oral gavage. Forty-eight mice were randomly assigned into four groups [LS: low protein diet mice administered *S. alactolyticus* solution, LC: administered PBS (phosphate buffer solution), NS: normal-protein mice administered with *S. alactolyticus* solution, and NC: normal-protein mice administered with PBS]. All mice were sacrificed 3 weeks after the end of treatment, and jejunal contents were collected. A total of 16 amino acids, including 9 non-essential amino acids and 5 essential amino acids, were detected using untargeted metabolomics by LC–MS (Table S5). For the mice fed low protein diet, the total abundance of both non-essential amino acids (8.64 × 10^10^ *vs.* 9.89 × 10^10^, *P* = 0.034) and essential amino acids (3.71 × 10^10^ *vs.* 4.83 × 10^10^, *P* = 0.042) in the LS group were significantly higher than the LC group. The non-essential amino acids (8.01 × 10^10^ vs. 8.72 × 10^10^, *P* = 0.21) and essential amino acids (3.37 × 10^10^ *vs.* 3.99 × 10^10^, *P* = 0.087) were higher in the NS group, but without statistical significance. In particular, the abundance of L-Valine (LC *vs.* LS: *P* = 0.039) and L-tyrosine (LC *vs.* LS: *P* = 0.027) were significantly higher in *S. alactolyticus* treatment groups for the low protein diet, while L-glutamic acid (NC *vs.* NS: *P* = 0.033) was significantly enriched in the treatment group (NS) when normal protein diet was fed.

## Discussion

As one of the “vulnerable of extinction animals,” the giant panda is a symbol of wildlife conservation. The metagenomic analysis provides insight into not only the microbial composition but also the metabolic potential of the gut microbiome in giant pandas. Several studies based on metagenomic data have suggested that the gut microbiome of giant pandas plays a crucial role in the energy metabolism, environmental adaptation, and health [7, 11, 31]. However, the sample sizes in these studies have been small; thus, bacterial composition and functions in giant pandas remain largely unknown. Hence, a comprehensive investigation of the gut metagenome with a large sample size is needed. In the current study, we filled several knowledge gaps in this field by (1) constructing a total of 408 MAGs, (2) performing a pairwise comparison of metagenome and meta-transcriptome to reveal the contribution of gut microbiota to host’s dietary changes by protein metabolism in giant pandas, and (3) identifying a key player (*S. alactolyticus*) of the panda’s gut microbiota and verifying its roles in the biosynthesis of essential AAs in a mice experiment.

The reconstruction of MAGs has become a common task for microbiome studies as it provides critical insights into the microbial diversity, composition, and functions of a specific niche. Therefore, several studies have reconstructed MAGs in many mammalian species, such as humans [32], mice [15], pigs [20], and chickens [22]. Despite the growing knowledge of gut metagenomes, little is known about the MAGs in giant pandas. Jin et al. [24] reconstructed bacterial genomes from 60 fecal samples, which resulted in only 22 “high quality” (completeness values ≥ 70% and contamination ≤ 10%). Another study [33] assembled 30 MAGs (called metagenome linking group in the original paper) with a higher quality (completeness values ≥ 80% and contamination ≤ 25%) from 57 samples with an average sequencing depth of 5.25 Gbp ranging from 1.5 to 9.7 Gbp. In this study, we recovered the largest number of MAGs from giant pandas to date (*n* = 408) by substantially expanding the sample size (*n* = 322) and sequencing depth using both next- and third-generation sequencing technologies. Of note, we sequenced 98 samples with a much greater sequencing depth (~ 20 Gbp/sample for captive, ~ 35 Gbp/sample for wild giant pandas) than previous studies in giant pandas as well as in recent studies focusing on MAGs construction in pigs (11.46 Gbp/sample) [20] and buffalo (6.2 Gbp/sample) [21]. In addition, to improve the continuity and completeness of MAGs, we also constructed five Oxford Nanopore Technologies (ONT) libraries and generated ONT reads of ~ 61 Gbp/pool sample, together with the Illumina sequencing, resulted in 148 near-complete MAGs. Published studies have suggested that the gut microbiome of giant pandas has a lower gut bacterial richness than other animals [10, 34, 35]. This study’s reconstruction of 408 medium- or high-quality MAGs remarkably increased the microbial richness in giant pandas compared to previous studies [24, 33].

Among the 408 MAGs, 206 were classified into 149 bacterial species from the Genome Database Taxonomy (GTDB), with *S. alactolyticus* as the most abundant MAG, consistent with findings in our previous study [10] that *Streptococcus* was the most abundant genus in the gut of captive giant panda. Jin’s study [24] identified *Streptococcus* as the second most abundant genus using a 16 s rRNA-based method. The discrepancy between these two studies might be due to the technology used to study the microbiome. Nevertheless, although *S. alactolyticus* has been reported as an opportunistic pathogen in humans [36], it has been isolated from many animals such as pigs [37, 38], chickens [39], dogs [40], cows [41], and pigeons [42]. Interestingly, the most abundant MAG in giant pandas in this study, MAG 212 and MAG 172, were also classified as *Streptococcus*, although species-level classification was not available for these MAGs. Of note, 202 of the 408 MAGs did not match any existing reference genomes in the GTDB, suggesting that our data substantially expanded reference genomes in the gut microbiome of giant pandas at the species level.

### Meta-transcriptomic data analysis provides novel insights into gene expression profiles involved in nutrient metabolism

Giant pandas feed almost exclusively on highly fibrous bamboo that contains a large amount of cellulose and hemicellulose, which, if completely digested, would provide additional energy from carbohydrates. They were not fed any commercial diets. Due to the lack of genes involved in cellulose degradation in the host, there has been a growing interest in the gut microbiota’s functions in cellulose metabolism. Several metagenomics studies have shown the presence of genes encoding enzymes involved in digesting cellulose [7], hemicellulose [33], and lignin [43] from the gut microbiota of giant pandas. However, our previous study showed that the gut microbiome of the giant panda was more similar to those of other carnivores and that the abundance of genes encoding cellulose and hemicellulose degradation enzymes was low [9]. In this study, we first confirmed this result by metagenome analyses of a large number of samples with great sequencing depth. We next examined the expression of cellulose and hemicellulose digesting genes by meta-transcriptomics data analysis from 14 captive giant pandas. Consistently, we found that the expression of those genes in giant pandas was also low, similar to that of the carnivore cat and lower than that of humans and pigs, suggesting that the gut microbiota of giant pandas was not efficiently helping its host utilize bamboo fibers for additional energy.

Bamboo is not only rich in fiber; it is also high in proteins, which provide about 61% of the energy in giant pandas, equivalent to that of the hypercarnivores as estimated by a macronutrient composition analysis [13]. We thus investigated the microbial metabolic pathways involved in amino acid degradation and biosynthesis in giant pandas. Metagenomic and meta-transcriptomic analyses show that the genes encoding amino acid metabolism in giant pandas were distinct from those of other mammals, suggesting the existence of a unique set of gene families involved in amino acid metabolism in giant pandas. Both the abundance and expression levels of these genes in the gut microbiome of giant pandas were more similar to those of the carnivores (e.g., cats) and were greater than those in omnivores and herbivores. This data suggest that bacteria were actively involved in protein metabolism as a major energy source for giant pandas.

Metagenomics and meta-transcriptomics are important approaches to studying the gut microbiota in different animal species. Metagenomics provides insights into the metabolic potential of the microbiota, revealing the abundance of the major bacterial taxa and metabolic pathways. Meta-transcriptomics, on the other hand, captures the active bacteria and their functions by analyzing the expressed genes. Members of the gut microbiota switch from dormant to active states frequently. Therefore, the most dominant bacterial species might not necessarily be the most active species. In addition, the genes encoding the metabolic pathways may or may not be transcribed, depending on many variables. Consequently, there are often differences in the overall gut microbiota and specific taxa revealed by metagenomics and meta-transcriptomics [44, 45]. In our study, we conducted metagenome and meta-transcriptome sequencing on the same set of samples to explore the abundant versus the “active” bacteria. It is worth noting that members of *E. flexneri* and *E. coli* have been associated with infectious diseases in humans and other animals, and their abundances were relatively high in pandas’ guts. However, the expression levels of these species were relatively low, and the pandas have been healthy, suggesting that these species were not that “active” and were not causing diseases. In addition, MAGs associated with *Escherichia* also possess genes involved in carbohydrate degradation. Therefore, it is difficult to draw any conclusions regarding their roles in pandas’ health or nutrition. Interestingly, *S. alactolyticus* (24.34%) was less abundant than *E. flexneri* (27.10%) based on the metagenomic data; however, its expression level was greater (45.45% *vs.* 10.49%), suggesting that *S. alactolyticus* may be an “active” member and plays a more prominent role in pandas’ gut microbiome.

### S. alactolyticus may contribute to the essential amino-acid synthesis

Our analyses showed that KEGG modules of essential amino-acid biosynthesis were significantly enriched in giant pandas with a higher relative abundance of *S. alactolyticus*, suggesting that *S. alactolyticus* may be an important factor affecting essential amino-acid biosynthesis in the gut microbiota of giant pandas. To establish a causal relationship, we subsequently fed mice with *S. alactolyticus.* We detected a greater amount of both non-essential and essential amino acids in the jejunum of mice, suggesting the involvement of *S. alactolyticus* in essential amino-acid biosynthesis. Previous studies indicated that *S. alactolyticus* was a lactic acid-producing bacterium associated with carbohydrate fermentation [40] and was considered a potential probiotic for chicken [39]. Giant pandas experienced a drastic dietary change from an animal protein diet (meat) to a plant protein diet (bamboo), making it difficult for them to meet the needs of essential amino acids. Our data showed the involvement of *S. alactolyticus* in amino-acid biosynthesis, suggesting this bacterium plays an important role in the host’s dietary adaptation to bamboos.

## Conclusion

In this study, we reconstructed a total of 408 MAGs. These data not only substantially improved our view of microbial diversity and metabolic potential but also provided a foundation for future metagenomics studies. In addition, we also performed a pairwise investigation of metagenomes and meta-transcriptomes in a subset of the samples, which allowed us to distinguish between abundant and active bacteria and determine gene expression profiles involved in nutrient metabolism. Our data show that the gut microbiota in the giant pandas plays important roles in protein rather than carbohydrate metabolism. *S. alactolyticus*, the most abundant bacterial species, were involved in the protein metabolism of the intestinal of giant pandas. The unique protein metabolic profiles in the gut microbiome discovered in this study, the straight and short carnivore-like gut intestinal tract, together with the evolutionary adaptations in their teeth, skull, and pseudo-thumb needed to process bamboo [46], explain, at least partially, the pandas’ dietary adaption to bamboos.

## Materials and methods

### Sample collection of giant pandas and cats

A total of 90 fecal samples were collected from 36 captive giant pandas at the Chengdu Research Base of Giant Panda Breeding and 54 captive giant pandas at China Research (Chengdu, China) and Conservation Center for the Giant Panda at Wolong (Wolong, China). In addition, eight fresh fecal samples of wild giant pandas collected from Wolong Nature Reserve (China) were also included in this study. All stool samples were flash-frozen in liquid nitrogen and stored at − 80 °C until further processing. We also included 13 fresh fecal samples from healthy cats from the cat feeding base at Remigao Animal Nutrition and Health Technology Co., Ltd. (Guangzhou, Guangdong, China). Fecal samples of cats were flash-frozen in liquid nitrogen and stored at − 80 °C until further processing.

### DNA and RNA extraction, library preparation, and sequencing

The total DNA was extracted from thawed feces of giant pandas and cats by using the Magnetic Universal Genomic DNA Kit (QIAGEN Inc., Beijing, China) according to the manufacturer’s protocol. The DNA quality and integrity were assessed on 1% agarose gels. DNA concentration was measured using Qubit® DNA Assay Kit with a Qubit® 3.0 Fluorometer (Invitrogen, China). Library preparation was performed using the NEB Next® Ultra™ DNA Library Prep Kit for Illumina (NEB, USA) following the manufacturer’s recommendations. The qualified DNA libraries were sequenced on the Illumina NovaSeq 6000 (PE150) platform.

The total RNA was extracted from the feces of giant pandas and cats using the RNAprep Pure Cell/Bacteria Kit (TIANGEN, Beijing, China). RNA quality and concentration were first examined using 1% agarose gels and a NanoPhotometer® spectrophotometer and then further confirmed with an RNA 6000 Nano Assay kit of the 2100 Bioanalyzer (Agilent Technologies, China) and Qubit® 3.0 Fluorometer (Invitrogen, USA). The ribosomal RNA was depleted from total RNA using TRIzol (Invitrogen, China) before meta-transcriptomic library construction, which was subjected to meta-transcriptomic sequencing on the Illumina NovaSeq 6000 (PE150) platform.

### Reconstruction and quality assessment of metagenomic assembled genomes (MAGs)

To construct comprehensive MAGs in giant pandas, we also downloaded additional shotgun metagenomic datasets of giant pandas from three previous studies by Wu et al. [27] (*n* = 27) and Guo et al. [25, 26] (*n* = 197) in addition to the deep sequencing data generated in our study, resulting in a total of 322 gut metagenomes. The Kneaddata pipeline v0.7.2 (https://bitbucket.org/biobakery/kneaddata) was applied for quality control and removal of host contamination. In brief, Trimmomatic v0.39 [47] in the pipeline was used to remove low-quality reads, and bowtie2 was applied to identify and remove host (GCF_002007445.1), and diet-contaminant (GCA_017311315.1 and GCA_011038535.1) reads [48].

The hybrid assembly of contigs from the ONT and Illumina clean reads was carried out using both OPERA-MS [32] pipeline and HybridSPAdes [28]. Clean reads of each sample were first assembled independently using the metaSpades [49] to obtain more complete gene scaffolds of the giant pandas. Next, the assembled scaffolds longer than 2000 bp were subjected to genome binning using MetaBAT2 [50] with default parameters. Bins longer than 1 M bp were selected for further analysis. The dRep software [51] was used to remove redundant bins with the parameter ‘-sa 0.99’. We used CheckM (lineage_wf) [52] to determine the final quality of bins, including completeness and contamination, and only kept MAGs with completeness > 50% and contamination < 10%. The tRNA and rRNA genes of MAGs were annotated using Barrnap (https://github.com/tseemann/barrnap) and tRNAscan-SE [53], respectively. The alignment rate of metagenomic reads to MAGs was measured using Bowtie2 software [48]. The taxonomy assignment of these MAGs was inferred using GTDB-TK v1.5.0 [54] with the GTDB reference (R06-RS202, March 21, 2022).

### Meta-transcriptomic data analysis to reveal active bacterial taxa and gene expression profiles

The raw meta-transcriptomic dataset, coming from giant panda and cat sequenced in this study, and the other five host species downloaded from public databases, were processed with the Kneaddata pipeline described in the section described above to perform quality control and host-contaminant removal. SortMeRNA software (v4.3.2) [55] and SMR v4.3 sensitive database were used to remove potential rRNA sequences from clean reads of both meta-transcriptomic and metagenomic samples.

### The measure of taxonomic diversity

To determine the abundant versus the active bacterial taxa in giant pandas, we performed a pairwise comparison of the metagenome and metatranscriptome data in the 14 giant pandas. To this end, we first built a customized database using the genome sequences of the 408 MAGs based on the format of the Kraken2 taxonomic classification software [56]. We then used Kraken2 to assign metagenomic and meta-transcriptomic reads to MAGs in the customized database to generate profiles of the abundant (based on metagenome data) and active (based on the metatranscriptome data) MAGs in each of the 14 giant pandas.

QIIME2 version 2021.4 [57] was used to rarefy the reads count table and calculate the relative abundance of MAGs in each sample. The alpha diversity (Shannon Index and Observed feature), beta diversity (Jaccard and Bray–Curtis), and related statistical analyses were also performed on the QIIME2 platform.

### Functional gene profiles involved in nutrient digestion

To compare the abundance and expression of cellulose degradation-related genes, and amino acid degradation-related genes in giant pandas, and place these data in the context of mammalian dietary patterns, we downloaded five datasets containing both gut metagenomes and metatranscriptomes in cattle (rumen, *n* = 14) [44], sheep (rumen, *n* = 10) [58], humans (feces, *n* = 13) [59], mice (feces, *n* = 8) [60], and pigs (feces, *n* = 6) [61].

For other host species (cat, human, mouse, pig, cattle, and sheep), contigs assembly and gene prediction were separately performed for each species. Both metagenomic and metatranscriptomic data were analyzed as follows. A non-redundant gene catalog of each species was generated by clustering with CD-HIT-EST [62] at a cutoff of 0.95 similarity. Gene functional annotation identification were achieved by using the eggnog-mapper [63]. Gene quantification was conducted using the Salmon Program [64]. Finally, different functional gene clusters were generated using customized Python scripts. The beta diversity of gut/rumen microbiome among different host species based on Bray–Curtis distance calculated using these gene profiles was measured on the QIIME2 platform (version 2021.4) [57]. Analysis of similarities (ANOSIM) was adapted to explore the differences in beta diversity between groups. For all analyses, statistical significance was determined at *P* ≤ 0.05. Except where noted, the figures were generated with the R package ggplot2.

### Isolation of S. alactolyticus from fecal samples of giant pandas

Seven fecal samples from giant pandas with a higher relative abundance of *S. alactolyticus*, according to metagenomic analysis, were pooled and diluted 100-fold in PBS. The diluted solution was streaked on a brain heart infusion (BHI, DSMZ Medium 215) agar plate. Agar plates were incubated anaerobically for 24 h at 37 °C. After that, colonies were picked and streaked on a new BHI agar plate for pure cultures. The isolated microbial colonies were used for DNA extraction, 16S rDNA sequencing using the Sanger sequencing method, and alignment by the online blast (https://blast.ncbi.nlm.nih.gov) to identify the taxonomy of these colonies. The colonies identified as *S. alactolyticus* were stored using a mixed solution of newborn calf serum (4%), glycerin (16%), and brain heart infusion (80%).

### Validation of the functions of S. alactolyticus in a mice experiment

A total of 48 BALB/cJ female mice aged 6 weeks were purchased from a commercial company (Novogene, Beijing, China). These mice were housed in standard cages (1 mouse per cage) under an average room temperature of 25℃ with corncob bedding. After 3 days of acclimation, all mice were randomly assigned into four groups (*n* = 12/group), including LC, LS, NC, and NS groups. LC and LS were fed a low protein diet (10% crude protein), and NC and NS groups were fed with a normal-protein diet (18% crude protein). The mice in the treatment group of LS and NS groups were administered with *S. alactolyticus* solution by gavage on day 1, day 3, day 5, and day 7. In contrast, the mice from LC and NC groups were administered with PBS (phosphate buffer solution) as a negative control. All mice were fed with about 70% of the daily diet requirements (2.6 g), which were calculated based on the data during the period of pre-feeding. On day 28, all mice were slaughtered, and the intestinal digesta were collected from the jejunum. The sample of jejunal digesta was placed into a 2-ml polyethylene tube and then stored in liquid nitrogen to snap-freeze immediately.

### Metabolite profiling analysis

Untargeted metabolomics of jejunal digesta was carried out using liquid chromatograph mass spectrometer (LC–MS/MS) method analyses by a commercial company (Novogene, Beijing, China). Firstly, jejunal digesta (100 mg) was grounded separately with liquid nitrogen; subsequently, the homogenate was resuspended with prechilled 80% methanol and 0.1% formic acid. The processed samples were incubated at 4 °C for 5 min, followed by high-speed centrifugation (15,000r/min) for 5 min at 4 °C. Afterward, the supernatant was collected and diluted to a final concentration of 53% methanol with LC–MS grade water. Finally, the prepared samples were subjected to the LC–MS system for untargeted metabolomic analysis using the vanquish UHPLC system (Thermo Fisher Scientific, Germany) and Orbitrap Q ACTIVETM HF mass spectrometer (Thermo Fisher Scientific). The raw data were furtherly processed using compound discoverer 3.1 (CD3.1, Thermo Fisher Scientific) to obtain the quantitation of metabolites.

### Supplementary Information

**Additional file 1.** 

## Data Availability

Sequence data generated in this study was uploaded to the NCBI SRA database. It can be accessed via the accession number PRJNA872265. The metadata for all samples included in this study is presented in Table S1.

## Abbreviations

MAG: Metagenome-assembled genome
ONT: Oxford Nanopore Technologies
ANOSIM: Analysis of similarities
PCoA: Principal Coordinate Analysis
