# Supplementary material for: The unique gut microbiome of giant pandas involved in protein metabolism contributes to the host’s dietary adaption to bamboo
Source: Microbiome. 2023 Aug 14;11:180. doi: 10.1186/s40168-023-01603-0 (PMC10424351; doi:10.1186/s40168-023-01603-0)
Supplement: Supplementary file 2 — Additional file 1. [file 40168_2023_1603_MOESM1_ESM.pdf]

Figure S1. The top 15 most abundant genera in the giant panda gut.

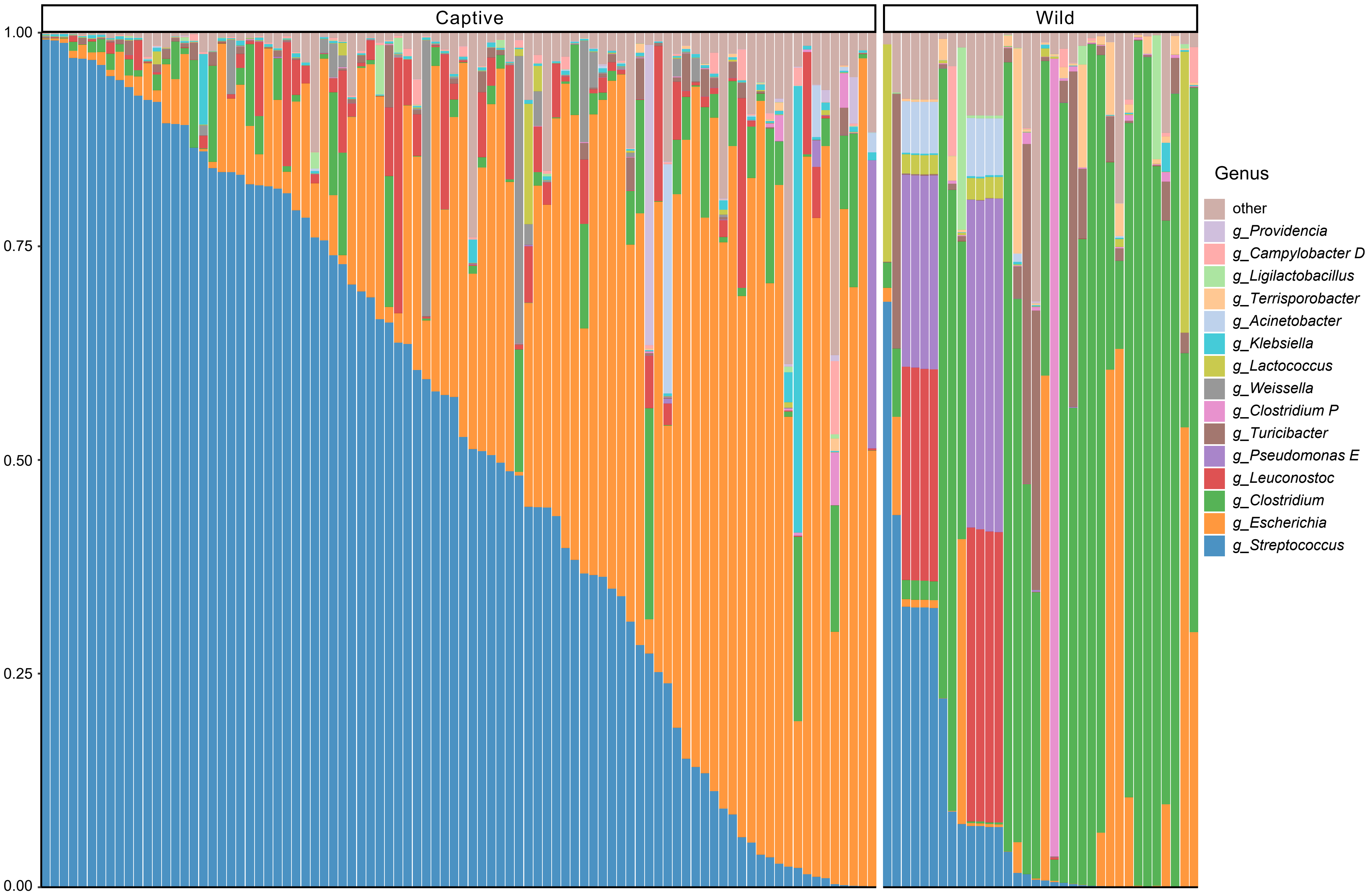

Figure S2. The top 15 most abundant MAGs in the giant panda gut

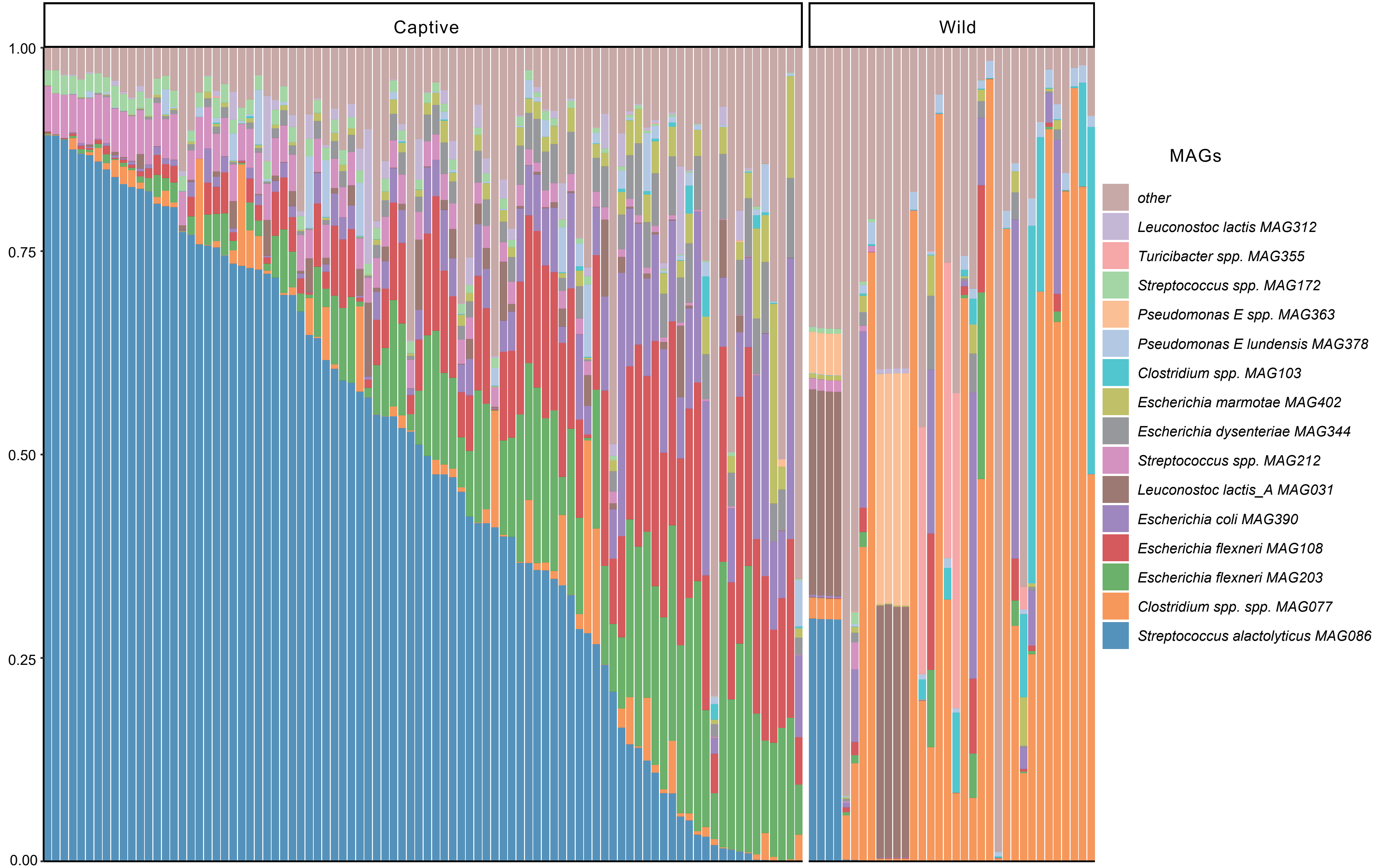

Figure S3. Composition of the top 10 species revealed by metagenomic abundance and metatranscriptomic expression at the species level.

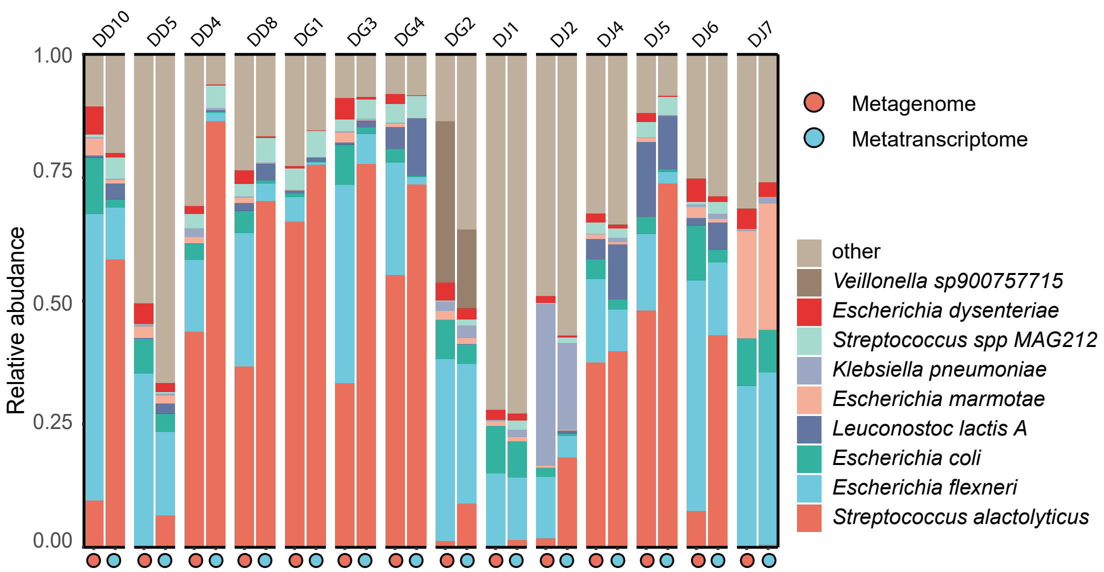

Table S1. Summary of sequenced and downloaded samples

Sample ID	Type of giant panda	Sex	Read length (bp)	# of reads	Sequencing depth (bp)	Accession Number
P1-160720-B	Captive	Male	100	146362573	29272514600	SRR7533009
P1-160720-V	Captive	Male	100	102209357	20441871400	SRR7533001
P1-160815-B1	Captive	Male	100	81148861	16229772200	SRR7532977
P1-160815-B2	Captive	Male	100	60585776	12117155200	SRR7532976
P1-160815-V	Captive	Male	100	173092735	34618547000	SRR7533000
P1-160817-B1	Captive	Male	100	94765687	18953137400	SRR7532983
P1-160817-B2	Captive	Male	100	30568063	6113612600	SRR7532982
P1-161107-B1	Captive	Male	100	89960322	17992064400	SRR7532888
P1-161107-B2	Captive	Male	100	70956218	14191243600	SRR7532889
P1-161107-V	Captive	Male	100	156096424	31219284800	SRR7533005
P1-161120-B1	Captive	Male	100	91927383	18385476600	SRR7532892
P1-161120-B2	Captive	Male	100	74818570	14963714000	SRR7532893
P1-161120-V	Captive	Male	100	88131620	17626324000	SRR7533004
P1-161215-B1	Captive	Male	100	73274430	14654886000	SRR7532884
P1-161215-B2	Captive	Male	100	85584870	17116974000	SRR7532885
P1-161215-V	Captive	Male	100	147488597	29497719400	SRR7533003
P1-170115-B1	Captive	Male	100	82702074	16540414800	SRR7532914
P1-170115-B2	Captive	Male	100	66973659	13394731800	SRR7532913
P1-170115-V	Captive	Male	100	136232416	27246483200	SRR7533007
P1-170215-B1	Captive	Male	100	57780316	11556063200	SRR7532910
P1-170215-B2	Captive	Male	100	112220534	22444106800	SRR7532909
P1-170215-V	Captive	Male	100	163994614	32798922800	SRR7533072
P1-170315-B1	Captive	Male	100	79812071	15962414200	SRR7533066
P1-170315-B2	Captive	Male	100	64972193	12994438600	SRR7533067
P1-170315-V	Captive	Male	100	151491991	30298398200	SRR7533070
P1-170416-B1	Captive	Male	100	88887539	17777507800	SRR7533030
P1-170416-B2	Captive	Male	100	86201466	17240293200	SRR7533028
P1-170416-V	Captive	Male	100	150415063	30083012600	SRR7532965
P1-170515-B1	Captive	Male	100	89729371	17945874200	SRR7532989
P1-170515-B2	Captive	Male	100	78449261	15689852200	SRR7532988
P1-170515-V	Captive	Male	100	164861581	32972316200	SRR7532896
P1-170616-B1	Captive	Male	100	83439067	16687813400	SRR7532932
P1-170616-B2	Captive	Male	100	97102354	19420470800	SRR7532933
P1-170616-V	Captive	Male	100	112530775	22506155000	SRR7532961
P1-170716-B1	Captive	Male	100	65067624	13013524800	SRR7532929
P1-170716-B2	Captive	Male	100	47675401	9535080200	SRR7532928
P1-170716-B3	Captive	Male	100	77712199	15542439800	SRR7532923
P1-170716-B4	Captive	Male	100	103535477	20707095400	SRR7532922
P1-170716-V	Captive	Male	100	112710202	22542040400	SRR7532959

Sample ID	Type of giant panda	Sex	Read length (bp)	# of reads	Sequencing depth (bp)	Accession Number
P-160607-BV1	Captive	Miss	100	99623404	19924680800	SRR7532973
P-160607-BV2	Captive	Miss	100	67672216	13534443200	SRR7532972
P2-160720-B1	Captive	Female	100	51128843	10225768600	SRR7532975
P2-160720-B2	Captive	Female	100	57947283	11589456600	SRR7532974
P2-160815-B1	Captive	Female	100	70187818	14037563600	SRR7532979
P2-160815-B2	Captive	Female	100	104639735	20927947000	SRR7532978
P2-160815-V	Captive	Female	100	127407781	25481556200	SRR7532999
P2-160817-B	Captive	Female	100	102054005	20410801000	SRR7532941
P2-160914-B1	Captive	Female	100	60869073	12173814600	SRR7532886
P2-160914-B2	Captive	Female	100	87180222	17436044400	SRR7532887
P2-160914-V	Captive	Female	100	129565918	25913183600	SRR7532998
P2-161115-B1	Captive	Female	100	95135528	19027105600	SRR7532890
P2-161115-B2	Captive	Female	100	77300745	15460149000	SRR7532891
P2-161215-B1	Captive	Female	100	93303364	18660672800	SRR7532916
P2-161215-B2	Captive	Female	100	89224569	17844913800	SRR7532915
P2-161215-V	Captive	Female	100	164913105	32982621000	SRR7533002
P2-170115-B1	Captive	Female	100	85444105	17088821000	SRR7532912
P2-170115-B2	Captive	Female	100	110133730	22026746000	SRR7532911
P2-170115-V	Captive	Female	100	141337316	28267463200	SRR7533006
P2-170215-B1	Captive	Female	100	78432723	15686544600	SRR7532918
P2-170215-B2	Captive	Female	100	54363481	10872696200	SRR7532917
P2-170215-V	Captive	Female	100	89945947	17989189400	SRR7533073
P2-170315-B1	Captive	Female	100	76391608	15278321600	SRR7533064
P2-170315-B2	Captive	Female	100	57207593	11441518600	SRR7533065
P2-170315-V	Captive	Female	100	82159626	16431925200	SRR7533071
P2-170416-B1	Captive	Female	100	91589369	18317873800	SRR7533026
P2-170416-B2	Captive	Female	100	97879896	19575979200	SRR7533025
P2-170416-V	Captive	Female	100	186266002	37253200400	SRR7532966
P2-170515-B1	Captive	Female	100	90506647	18101329400	SRR7532934
P2-170515-B2	Captive	Female	100	62891403	12578280600	SRR7532935
P2-170515-V	Captive	Female	100	84169101	16833820200	SRR7532964
P2-170615-B1	Captive	Female	100	53183379	10636675800	SRR7532968
P2-170615-B2	Captive	Female	100	58014825	11602965000	SRR7532969
P2-170615-B3	Captive	Female	100	65965691	13193138200	SRR7532927
P2-170615-B4	Captive	Female	100	55752303	11150460600	SRR7532926
P2-170615-V	Captive	Female	100	105326243	21065248600	SRR7532962
P2-170716-B1	Captive	Female	100	54496464	10899292800	SRR7532925
P2-170716-B2	Captive	Female	100	41268034	8253606800	SRR7532924
P2-170716-B3	Captive	Female	100	57463800	11492760000	SRR7532931
P2-170716-B4	Captive	Female	100	40256057	8051211400	SRR7532930
P2-170716-V	Captive	Female	100	171074233	34214846600	SRR7532960

Sample ID	Type of giant panda	Sex	Read length (bp)	# of reads	Sequencing depth (bp)	Accession Number
P3-160627-BV	Captive	Female	100	170758698	34151739600	SRR7533077
P3-160704-BV1	Captive	Female	100	78522741	15704548200	SRR7533062
P3-160704-BV2	Captive	Female	100	48969708	9793941600	SRR7533063
P3-160713-BV	Captive	Female	100	169043569	33808713800	SRR7533075
P3-160803-BV	Captive	Female	100	141579057	28315811400	SRR7533068
P3-160809-BV	Captive	Female	100	161092138	32218427600	SRR7533051
P3-160815-BV1	Captive	Female	100	41799448	8359889600	SRR7533087
P3-160815-BV2	Captive	Female	100	73146761	14629352200	SRR7533086
P3-160821-BV1	Captive	Female	100	129575315	25915063000	SRR7533083
P3-160821-BV2	Captive	Female	100	107117742	21423548400	SRR7533082
P3-160827-BV	Captive	Female	100	84759195	16951839000	SRR7533050
P3-161011-BV	Captive	Female	100	147069904	29413980800	SRR7533055
P3-161213-BV	Captive	Female	100	186483193	37296638600	SRR7533054
P3-170118-BV1	Captive	Female	100	81044907	16208981400	SRR7533015
P3-170118-BV2	Captive	Female	100	52270249	10454049800	SRR7533016
P3-170219-BV1	Captive	Female	100	48555820	9711164000	SRR7533013
P3-170219-BV2	Captive	Female	100	73332792	14666558400	SRR7533014
P3-170319-BV1	Captive	Female	100	54582000	10916400000	SRR7533020
P3-170319-BV2	Captive	Female	100	119907782	23981556400	SRR7533021
P4-160626-BV	Captive	Female	100	172214184	34442836800	SRR7533076
P4-160627-BV	Captive	Female	100	114932138	22986427600	SRR7533074
P4-160704-BV1	Captive	Female	100	68874081	13774816200	SRR7533060
P4-160704-BV2	Captive	Female	100	67218301	13443660200	SRR7533061
P4-160713-BV1	Captive	Female	100	83214584	16642916800	SRR7533058
P4-160713-BV2	Captive	Female	100	55913016	11182603200	SRR7533059
P4-160803-BV	Captive	Female	100	124085718	24817143600	SRR7533069
P4-160809-BV1	Captive	Female	100	70797017	14159403400	SRR7533085
P4-160809-BV2	Captive	Female	100	105075468	21015093600	SRR7533084
P4-160815-BV1	Captive	Female	100	54263637	10852727400	SRR7533081
P4-160815-BV2	Captive	Female	100	37822882	7564576400	SRR7533080
P4-160821-BV1	Captive	Female	100	91754495	18350899000	SRR7533079
P4-160821-BV2	Captive	Female	100	79331019	15866203800	SRR7533078
P4-160827-BV	Captive	Female	100	162810496	32562099200	SRR7533053
P4-160828-BV	Captive	Female	100	147472278	29494455600	SRR7532940
P4-160915-BV	Captive	Female	100	144576624	28915324800	SRR7533052
P4-161214-BV	Captive	Female	100	172880031	34576006200	SRR7533057
P4-170118-BV1	Captive	Female	100	80638856	16127771200	SRR7533017
P4-170118-BV2	Captive	Female	100	76867448	15373489600	SRR7533018
P4-170214-BV1	Captive	Female	100	81894833	16378966600	SRR7533011
P4-170214-BV2	Captive	Female	100	73890119	14778023800	SRR7533012
P4-170319-BV1	Captive	Female	100	106180311	21236062200	SRR7533038

Sample ID	Type of giant panda	Sex	Read length (bp)	# of reads	Sequencing depth (bp)	Accession Number
P4-170319-BV2	Captive	Female	100	72950711	14590142200	SRR7533037
R1-161113-B1	Captive	Miss	100	61285855	12257171000	SRR7533036
R1-161113-B2	Captive	Miss	100	46899218	9379843600	SRR7533035
R1-161113-V	Captive	Miss	100	171077905	34215581000	SRR7533056
R1-161120-B1	Captive	Miss	100	51223262	10244652400	SRR7532905
R1-161120-B2	Captive	Miss	100	81292305	16258461000	SRR7532895
R1-161120-V	Captive	Miss	100	134001607	26800321400	SRR7532945
R1-161129-B1	Captive	Miss	100	76633575	15326715000	SRR7533040
R1-161129-B2	Captive	Miss	100	52404765	10480953000	SRR7533039
R1-161129-V	Captive	Miss	100	90855453	18171090600	SRR7533047
R1-161208-B1	Captive	Miss	100	50156323	10031264600	SRR7532952
R1-161208-B2	Captive	Miss	100	56486883	11297376600	SRR7532953
R1-161208-V	Captive	Miss	100	161633748	32326749600	SRR7532902
R1-161215-B1	Captive	Miss	100	60592277	12118455400	SRR7532950
R1-161215-B2	Captive	Miss	100	92040376	18408075200	SRR7532951
R1-161215-V	Captive	Miss	100	162603362	32520672400	SRR7533090
R1-170115-B1	Captive	Miss	100	88879579	17775915800	SRR7532954
R1-170115-B2	Captive	Miss	100	62820500	12564100000	SRR7532955
R1-170115-V	Captive	Miss	100	154110390	30822078000	SRR7532907
R1-170215-B1	Captive	Miss	100	70833306	14166661200	SRR7532991
R1-170215-B2	Captive	Miss	100	51129179	10225835800	SRR7532990
R1-170315-B1	Captive	Miss	100	61364355	12272871000	SRR7532997
R1-170315-B2	Captive	Miss	100	49960236	9992047200	SRR7532996
R1-170315-B3	Captive	Miss	100	61685287	12337057400	SRR7532987
R1-170315-B4	Captive	Miss	100	48490638	9698127600	SRR7532986
R1-170315-V	Captive	Miss	100	169400469	33880093800	SRR7532899
R1-170416-B1	Captive	Miss	100	87036752	17407350400	SRR7532894
R1-170416-B2	Captive	Miss	100	94485362	18897072400	SRR7532900
R1-170416-V	Captive	Miss	100	219756148	43951229600	SRR7533099
R1-170516-B1	Captive	Miss	100	94639518	18927903600	SRR7532919
R1-170516-B2	Captive	Miss	100	85212171	17042434200	SRR7533049
R1-170516-V	Captive	Miss	100	159337447	31867489400	SRR7533096
R1-170615-B1	Captive	Miss	100	81487667	16297533400	SRR7533089
R1-170615-B2	Captive	Miss	100	101634539	20326907800	SRR7533023
R1-170615-V	Captive	Miss	100	159006642	31801328400	SRR7533094
R1-170716-B1	Captive	Miss	100	79193298	15838659600	SRR7533033
R1-170716-B2	Captive	Miss	100	60225836	12045167200	SRR7533022
R1-170716-V	Captive	Miss	100	124610693	24922138600	SRR7533092
R2-161113-B1	Captive	Miss	100	58053171	11610634200	SRR7533042
R2-161113-B2	Captive	Miss	100	28170400	5634080000	SRR7533041
R2-161113-V	Captive	Miss	100	182569131	36513826200	SRR7533048

Sample ID	Type of giant panda	Sex	Read length (bp)	# of reads	Sequencing depth (bp)	Accession Number
R2-161120-B1	Captive	Miss	100	52014768	10402953600	SRR7533031
R2-161120-B2	Captive	Miss	100	63799469	12759893800	SRR7533088
R2-161120-V	Captive	Miss	100	110561057	22112211400	SRR7533034
R2-161129-B1	Captive	Miss	100	63790057	12758011400	SRR7533045
R2-161129-B2	Captive	Miss	100	50182109	10036421800	SRR7533044
R2-161129-V	Captive	Miss	100	195373959	39074791800	SRR7532901
R2-161208-B	Captive	Miss	100	84491604	16898320800	SRR7532903
R2-161208-V	Captive	Miss	100	234061574	46812314800	SRR7532904
R2-161215-B1	Captive	Miss	100	54211293	10842258600	SRR7532956
R2-161215-B2	Captive	Miss	100	120668043	24133608600	SRR7532957
R2-161215-V	Captive	Miss	100	147542421	29508484200	SRR7532906
R2-170115-B1	Captive	Miss	100	80342280	16068456000	SRR7532948
R2-170115-B2	Captive	Miss	100	67885860	13577172000	SRR7532949
R2-170115-V	Captive	Miss	100	73628934	14725786800	SRR7532908
R2-170215-B1	Captive	Miss	100	2.27E+07	4542309200	SRR7532993
R2-170215-B2	Captive	Miss	100	44681373	8936274600	SRR7532992
R2-170215-B3	Captive	Miss	100	42056956	8411391200	SRR7532995
R2-170215-B4	Captive	Miss	100	28782185	5756437000	SRR7532994
R2-170215-V	Captive	Miss	100	179611454	35922290800	SRR7532898
R2-170315-B1	Captive	Miss	100	35691149	7138229800	SRR7532967
R2-170315-B2	Captive	Miss	100	23101180	4620236000	SRR7532963
R2-170315-B3	Captive	Miss	100	66462651	13292530200	SRR7532981
R2-170315-B4	Captive	Miss	100	66819956	13363991200	SRR7532980
R2-170315-V	Captive	Miss	100	109457139	21891427800	SRR7533100
R2-170416-B	Captive	Miss	100	164965175	32993035000	SRR7533098
R2-170416-V	Captive	Miss	100	120833125	24166625000	SRR7533097
R2-170516-B1	Captive	Miss	100	78052536	15610507200	SRR7532920
R2-170516-B2	Captive	Miss	100	57539887	11507977400	SRR7532921
R2-170516-V	Captive	Miss	100	88487376	17697475200	SRR7533095
R2-170615-B1	Captive	Miss	100	69508393	13901678600	SRR7532958
R2-170615-B2	Captive	Miss	100	53773894	10754778800	SRR7533024
R2-170615-V	Captive	Miss	100	147730607	29546121400	SRR7533093
R2-170716-B1	Captive	Miss	100	134426928	26885385600	SRR7533019
R2-170716-B2	Captive	Miss	100	107992844	21598568800	SRR7533008
R2-170716-V	Captive	Miss	100	191385929	38277185800	SRR7533091
CC1301	Wild	Male	101	25451795	5141262590	CRR055205
CY1204	Wild	Male	101	34701556	7009714312	CRR055206
CY1205	Wild	Male	101	42370946	8558931092	CRR055207
CY1305	Wild	Male	101	32371116	6538965432	CRR055208
CY1307	Wild	Male	101	36719938	7417427476	CRR055209
CY1208	Wild	Male	101	44348437	8958384274	CRR055210

Sample ID	Type of giant panda	Sex	Read length (bp)	# of reads	Sequencing depth (bp)	Accession Number
CY1308	Wild	Male	101	42079035	8499965070	CRR055211
NN1201	Wild	Female	101	28795050	5816600100	CRR055212
NN1204	Wild	Female	101	32345938	6533879476	CRR055213
NN1301	Wild	Female	101	37471094	7569160988	CRR055214
NN1303	Wild	Female	101	18032938	3642653476	CRR055215
NN1305	Wild	Female	101	30783431	6218253062	CRR055216
WW1208	Wild	Male	101	40250417	8130584234	CRR055217
WW1307	Wild	Male	101	32889255	6643629510	CRR055218
WW1308	Wild	Male	101	36147084	7301710968	CRR055219
XM1303	Wild	Female	101	23602751	4767755702	CRR055220
XM1305	Wild	Female	101	41872462	8458237324	CRR055221
XM1307	Wild	Female	101	35038011	7077678222	CRR055222
XM1308	Wild	Female	101	37709153	7617248906	CRR055223
XY1201	Wild	Male	101	28336528	5723978656	CRR055224
XY1204	Wild	Male	101	27142142	5482712684	CRR055225
XY1207	Wild	Male	101	36158757	7304068914	CRR055226
XY1301	Wild	Male	101	17356961	3506106122	CRR055227
XY1303	Wild	Male	101	34051351	6878372902	CRR055228
XY1305	Wild	Male	101	33129779	6692215358	CRR055229
XY1307	Wild	Male	101	28503099	5757625998	CRR055230
XY1308	Wild	Male	101	34560390	6981198780	CRR055231
H-10	Captive	Female	150	70487899	21146369700	NA
H-1	Captive	Female	150	75597013	22679103900	NA
H-2	Captive	Male	150	68604229	20581268700	NA
H-3	Captive	Female	150	71028905	21308671500	NA
H-4	Captive	Female	150	68610058	20583017400	NA
H-5	Captive	Miss	150	80825469	24247640700	NA
H-6	Captive	Female	150	75167493	22550247900	NA
H-7	Captive	Female	150	68316383	20494914900	NA
H-8	Captive	Female	150	70055366	21016609800	NA
H-9	Captive	Female	150	68614227	20584268100	NA
SF-10	Captive	Female	150	68714678	20614403400	NA
SF-11	Captive	Female	150	101920153	30576045900	NA
SF-12	Captive	Male	150	71171705	21351511500	NA
SF-13	Captive	Female	150	66924514	20077354200	NA
SF-14	Captive	Female	150	67827031	20348109300	NA
SF-15	Captive	Female	150	72873789	21862136700	NA
SF-16	Captive	Female	150	70081251	21024375300	NA
SF-17	Captive	Female	150	79967451	23990235300	NA
SF-18	Captive	Female	150	72285143	21685542900	NA
SF-1	Captive	Male	150	68985546	20695663800	NA

Sample ID	Type of giant panda	Sex	Read length (bp)	# of reads	Sequencing depth (bp)	Accession Number
SF-2	Captive	Male	150	67179351	20153805300	NA
SF-3	Captive	Female	150	69781744	20934523200	NA
SF-4	Captive	Male	150	69447080	20834124000	NA
SF-5	Captive	Female	150	71895981	21568794300	NA
SF-6	Captive	Female	150	69228030	20768409000	NA
SF-7	Captive	Male	150	73679463	22103838900	NA
SF-8	Captive	Female	150	69228030	20768409000	NA
SN-10	Captive	Male	150	70179831	21053949300	NA
SN-11	Captive	Female	150	71294604	21388381200	NA
SN-13	Captive	Female	150	69510693	20853207900	NA
SN-14	Captive	Male	150	78948301	23684490300	NA
SN-15	Captive	Female	150	77087144	23126143200	NA
SN-16	Captive	Female	150	73007449	21902234700	NA
SN-1	Captive	Male	150	67487682	20246304600	NA
SN-2	Captive	Male	150	69920027	20976008100	NA
SN-3	Captive	Female	150	68362998	20508899400	NA
SN-4	Captive	Male	150	69801448	20940434400	NA
SN-5	Captive	Female	150	73545105	22063531500	NA
SN-6	Captive	Female	150	75647340	22694202000	NA
SN-8	Captive	Female	150	82136168	24640850400	NA
SN-9	Captive	Female	150	68813373	20644011900	NA
SW-10	Captive	Male	150	71868283	21560484900	NA
SW-11	Captive	Female	150	68954630	20686389000	NA
SW-12	Captive	Female	150	69543896	20863168800	NA
SW-14	Captive	Male	150	67043365	20113009500	NA
SW-15	Captive	Male	150	84590857	25377257100	NA
SW-1	Captive	Male	150	71265889	21379766700	NA
SW-2	Captive	Female	150	67740117	20322035100	NA
SW-3	Captive	Male	150	71679475	21503842500	NA
SW-5	Captive	Male	150	67496251	20248875300	NA
SW-6	Captive	Male	150	72672607	21801782100	NA
SW-7	Captive	Male	150	71385916	21415774800	NA
SW-8	Captive	Female	150	75203768	22561130400	NA
SW-9	Captive	Male	150	71770235	21531070500	NA
DD-1	Captive	Male	150	104801132	31440339600	NA
DD-10	Captive	Female	150	102144147	30643244100	NA
DD-11	Captive	Male	150	73596570	22078971000	NA
DD-2	Captive	Male	150	103392856	31017856800	NA
DD-3	Captive	Female	150	70253859	21076157700	NA
DD-4	Captive	Male	150	109349000	32804700000	NA
DD-5	Captive	Male	150	75712426	22713727800	NA

Sample ID	Type of giant panda	Sex	Read length (bp)	# of reads	Sequencing depth (bp)	Accession Number
DD-6	Captive	Female	150	69664963	20899488900	NA
DD-7	Captive	Female	150	85800657	25740197100	NA
DD-8	Captive	Female	150	107680392	32304117600	NA
DD-9	Captive	Male	150	80010565	24003169500	NA
DG-1	Captive	Female	150	106645933	31993779900	NA
DG-2	Captive	Male	150	69946604	20983981200	NA
DG-3	Captive	Male	150	103764943	31129482900	NA
DG-4	Captive	Male	150	102376492	30712947600	NA
DG-5	Captive	Female	150	110134695	33040408500	NA
DG-6	Captive	Male	150	67125806	20137741800	NA
DJ-1	Captive	Female	150	103092166	30927649800	NA
DJ-2	Captive	Male	150	111271028	33381308400	NA
DJ-3	Captive	Female	150	85829533	25748859900	NA
DJ-4	Captive	Male	150	100374185	30112255500	NA
DJ-5	Captive	Male	150	111527041	33458112300	NA
DJ-6	Captive	Female	150	103514515	31054354500	NA
DJ-7	Captive	Male	150	109228707	32768612100	NA
DP-1	Captive	Female	150	75444206	22633261800	NA
DP-2	Captive	Female	150	73876642	22162992600	NA
DP-3	Captive	Female	150	77093327	23127998100	NA
DP-4	Captive	Female	150	72973326	21891997800	NA
DP-5	Captive	Male	150	75102820	22530846000	NA
DS-1	Captive	Male	150	66945332	20083599600	NA
DS-2	Captive	Male	150	70067707	21020312100	NA
DS-3	Captive	Female	150	70442364	21132709200	NA
DS-4	Captive	Female	150	78492172	23547651600	NA
DS-5	Captive	Female	150	68907097	20672129100	NA
DS-6	Captive	Female	150	71326858	21398057400	NA
DS-7	Captive	Female	150	69151454	20745436200	NA
WY112	Wild	Miss	150	102395287	30718586100	NA
WY113	Wild	Miss	150	96649301	28994790300	NA
WY115	Wild	Miss	150	103728975	31118692500	NA
WY116	Wild	Miss	150	102394790	30718437000	NA
WY118	Wild	Miss	150	104707959	31412387700	NA
WY120	Wild	Miss	150	118740778	35622233400	NA
WY135	Wild	Miss	150	96558799	28967639700	NA
WY137	Wild	Miss	150	101824240	30547272000	NA

Table S2. Details of assembled medium- and high- quality MAGs

MAGs ID	genus	species	Completeness (%)	Contamination (%)	Num. of scaffolds	N50 of scaffolds (bp)	Num. of 16S rRNA	Num. of 5S rRNA	Num. of 23S rRNA	Num. of rRNA	Genes for Hemi/Cellulose
MAG001	g__Acinetobacter	s__Acinetobacter nosocomialis	100.00	1.94	1	3995565	3	3	3	9	0
MAG002	g__Basfia_A	s__MAG002	100.00	1.13	12	274234	0	0	0	0	0
MAG003	g__Brevundimonas	s__MAG003	100.00	0.65	19	460440	0	1	1	0	2
MAG004	g__Lactococcus	s__Lactococcus lactis_E	100.00	0.57	9	514361	0	1	0	0	1
MAG005	g__MAG005	s__MAG005	100.00	0.54	24	240186	0	0	0	0	0
MAG006	g__Bifidobacterium	s__Bifidobacterium breve	100.00	0.00	6	538961	0	0	0	0	1
MAG007	g__Terrisporobacter	s__Terrisporobacter mayombei	100.00	0.00	98	39038	0	3	0	2	1
MAG008	g__Basfia_A	s__MAG008	99.96	0.56	18	292715	0	1	0	0	0
MAG009	g__Polynucleobacter	s__MAG009	99.87	0.31	5	515287	0	1	0	0	0
MAG010	g__Veillonella	s__Veillonella sp900757715	99.85	0.00	27	110087	0	2	0	1	0
MAG011	g__Erysipelothrix	s__MAG011	99.76	0.00	86	46805	0	0	0	1	0
MAG012	g__Streptococcus	s__MAG012	99.64	1.03	27	163239	0	0	0	1	0
MAG013	g__Enterococcus_B	s__Enterococcus_B hirae	99.63	0.12	1	2705424	4	4	4	3	0
MAG014	g__Enterococcus	s__Enterococcus faecalis	99.63	0.00	7	386622	0	0	0	0	1
MAG015	g__Brumimicrobium	s__MAG015	99.46	2.70	89	70885	0	0	0	0	0
MAG016	g__Providencia	s__Providencia alcalifaciens	99.46	0.54	1	3782785	5	5	4	6	0
MAG017	g__Kocuria	s__MAG017	99.34	1.32	89	63030	0	0	0	0	0
MAG018	g__Enterococcus_J	s__MAG018	99.34	0.33	6	597069	0	0	0	0	0
MAG019	g__Enterococcus_G	s__Enterococcus_G sulfureus	99.34	0.00	1	2278706	3	3	3	19	1
MAG020	g__Ligilactobacillus	s__Ligilactobacillus animalis	99.30	0.00	6	373240	1	1	0	0	0
MAG021	g__Myroides	s__MAG021	99.26	9.70	94	69568	0	0	0	0	0
MAG022	g__Mycetocola	s__MAG022	99.24	0.34	1	3087133	5	6	5	0	1
MAG023	g__Wohlfahrtiimonas	s__MAG023	99.22	0.15	51	72325	0	0	0	0	0
MAG024	g__Enterococcus_D	s__Enterococcus_D sp00285[illegible]	99.12	0.38	1	3722498	4	4	4	3	4
MAG025	g__Lactobacillus	s__Lactobacillus johnsonii	99.03	0.00	7	342092	0	0	0	0	0
MAG026	g__Acinetobacter	s__Acinetobacter soli	99.00	2.52	11	708335	7	6	7	0	0
MAG027	g__Lactobacillus	s__Lactobacillus acidophilus	98.94	0.32	9	368600	0	0	0	0	0
MAG028	g__UBA3376	s__MAG028	98.92	2.21	156	22742	0	1	0	0	3
MAG029	g__Oceanisphaera	s__MAG029	98.89	0.00	54	81369	0	0	0	0	0
MAG030	g__Sphingobacterium	s__MAG030	98.89	0.79	37	119594	0	1	0	2	0
MAG031	g__Leuconostoc	s__Leuconostoc lactis_A	98.88	0.00	5	553628	0	0	0	0	0

MAGs ID	genus	species	Completeness (%)	Contamination (%)	Num. of scaffolds	N50 of scaffolds (bp)	Num. of 16S rRNA	Num. of 5S rRNA	Num. of 23S rRNA	Num. of rRNA	Genes for Hemi/Cellulose
MAG032	g__Lactococcus	s__Lactococcus garvieae	98.83	0.84	1	2163881	0	1	0	14	0
MAG033	g__Ligilactobacillus	s__MAG033	98.69	0.00	11	289587	0	1	0	0	0
MAG034	g__Anaerosporobacter	s__MAG034	98.66	3.80	171	38345	0	0	0	0	6
MAG035	g__Limosilactobacillus	s__Limosilactobacillus mucosa	98.64	0.00	1	2138120	2	2	2	18	0
MAG036	g__Enterococcus_C	s__Enterococcus_C saigonensi	98.60	1.11	105	45208	0	0	0	4	1
MAG037	g__Staphylococcus	s__MAG037	98.55	8.78	107	39215	0	1	0	1	3
MAG038	g__Frederiksenia	s__MAG038	98.52	2.91	79	97509	0	0	0	2	1
MAG039	g__Enterococcus_I	s__Enterococcus_I alcedinis	98.51	0.00	8	657615	0	0	0	0	0
MAG040	g__Sphingobacterium	s__Sphingobacterium kitahiros	98.46	1.61	103	113592	0	0	0	1	3
MAG041	g__Clostridium_P	s__Clostridium_P ventriculi	98.39	0.48	27	154413	0	1	0	2	0
MAG042	g__Providencia	s__Providencia alcalifaciens	98.37	0.00	14	573209	0	0	0	0	0
MAG043	g__Carnobacterium	s__Carnobacterium maltaroma	98.36	5.66	139	44518	0	0	0	2	1
MAG044	g__Lactococcus	s__Lactococcus lactis_E	98.33	2.26	3	1444583	2	3	2	12	1
MAG045	g__Ligilactobacillus	s__Ligilactobacillus salivarius	98.32	1.57	1	1609992	2	3	2	12	0
MAG046	g__Leucobacter	s__MAG046	98.25	0.58	41	120633	0	0	0	0	1
MAG047	g__Streptococcus	s__MAG047	98.20	0.00	13	222664	0	0	0	0	0
MAG048	g__Limosilactobacillus	s__Limosilactobacillus vaginal	98.19	0.54	42	64055	0	1	0	2	0
MAG049	g__Lactococcus	s__Lactococcus lactis	98.11	0.38	1	2133408	5	5	5	4	0
MAG050	g__Streptococcus	s__Streptococcus orisratti	98.08	1.16	1	2600539	6	6	7	0	1
MAG051	g__Pantoea	s__Pantoea brenneri	98.06	1.29	91	76461	0	0	0	0	2
MAG052	g__Paenibacillus	s__MAG052	98.04	1.29	30	433594	8	8	8	3	12
MAG053	g__UBA2632	s__MAG053	97.97	0.82	161	41804	0	0	0	0	2
MAG054	g__Hafnia	s__Hafnia alvei	97.90	0.88	47	182233	0	0	0	0	2
MAG055	g__Enterococcus	s__Enterococcus faecalis	97.88	0.37	1	2694999	2	2	2	6	1
MAG056	g__Brochothrix	s__Brochothrix thermosphacta	97.79	1.43	162	31719	0	0	0	2	2
MAG057	g__Weissella	s__Weissella confusa	97.78	2.91	1	2174119	6	7	6	9	0
MAG058	g__Flavobacterium	s__MAG058	97.76	4.35	200	35325	0	0	0	0	0
MAG059	g__Bifidobacterium	s__Bifidobacterium animalis	97.62	0.00	1	2239137	2	2	2	0	3
MAG060	g__Helicobacter_A	s__MAG060	97.51	0.82	30	100005	0	0	0	0	0
MAG061	g__Lactococcus	s__Lactococcus garvieae	97.39	1.15	93	32235	0	1	0	3	0
MAG062	g__Bacteroides	s__MAG062	97.37	0.64	92	47403	0	0	0	0	3
MAG063	g__Streptococcus	s__Streptococcus ferus	97.35	0.75	3	1147145	4	4	4	3	0
MAG064	g__Lactococcus	s__Lactococcus garvieae	97.33	2.91	1	2014915	5	5	5	1	0

MAGs ID	genus	species	Completeness (%)	Contamin ation (%)	Num. of scaffolds	N50 of scaffolds (bp)	Num. of 16S rRNA	Num. of 5S rRNA	Num. of 23S rRNA	Num. of rRNA	Genes for Hemi/Cellulo se
MAG065	g__Sphingobacterium	s__MAG065	97.33	3.81	101	79592	0	0	0	1	3
MAG066	g__Plesiomonas	s__Plesiomonas shigelloides	97.31	0.54	18	331152	0	0	0	0	1
MAG067	g__Klebsiella	s__Klebsiella quasipneumonia	97.29	5.76	20	546819	0	0	0	0	2
MAG068	g__Algoriella	s__Algoriella xinjiangensis	97.25	0.00	36	156837	0	0	0	0	3
MAG069	g__Lactococcus_A	s__Lactococcus_A laudensis	97.16	2.33	6	675684	1	1	1	3	0
MAG070	g__Streptococcus	s__Streptococcus pluranimaliu	97.09	0.37	19	158045	0	0	0	0	0
MAG071	g__Sphingobacterium	s__MAG071	97.00	0.16	61	109127	0	0	0	0	2
MAG072	g__Pseudomonas_D	s__MAG072	96.98	1.32	54	120304	0	0	0	0	0
MAG073	g__Soonwooa	s__MAG073	96.94	0.49	188	26177	0	0	0	0	2
MAG074	g__Streptococcus	s__Streptococcus mutans	96.88	0.87	45	62771	0	0	0	1	0
MAG075	g__Rodentibacter	s__Rodentibacter ratti	96.88	0.64	86	39416	0	0	0	1	0
MAG076	g__Bergeyella_A	s__MAG076	96.77	1.61	119	20437	0	0	0	2	0
MAG077	g__Clostridium	s__MAG077	96.77	0.81	101	39495	0	0	0	0	0
MAG078	g__Enterococcus_D	s__Enterococcus_D gallinarun	96.68	0.38	94	61244	0	0	0	2	1
MAG079	g__Clostridium_P	s__Clostridium_P massiliamaz	96.64	3.06	68	98950	0	2	0	1	0
MAG080	g__Providencia	s__Providencia alcalifaciens	96.63	3.80	64	177474	0	2	0	1	0
MAG081	g__Helicobacter_C	s__MAG081	96.60	1.71	107	31960	0	0	0	0	0
MAG082	g__Paenibacillus	s__MAG082	96.57	1.16	241	32514	0	1	0	0	5
MAG083	g__Pseudomonas_D	s__MAG083	96.56	0.60	56	86712	0	1	0	1	0
MAG084	g__Lactococcus	s__Lactococcus lactis	96.26	2.18	1	2386124	1	2	1	12	0
MAG085	g__Paenibacillus_C	s__MAG085	96.15	2.54	30	241474	5	6	5	3	10
MAG086	g__Streptococcus	s__Streptococcus alactolyticus	96.10	5.31	1	1769744	3	3	3	14	0
MAG087	g__Lactococcus	s__Lactococcus petauri	96.09	3.05	8	1263436	5	5	5	1	0
MAG088	g__Weissella	s__Weissella cibaria	96.06	2.49	1	2664304	7	8	7	0	0
MAG089	g__Necropsobacter	s__Necropsobacter rosorum	96.05	0.56	21	211580	0	0	0	0	0
MAG090	g__Weissella	s__Weissella confusa	96.01	0.00	1	2103398	6	7	6	9	0
MAG091	g__CAIQBG01	s__MAG091	95.95	0.26	30	103305	0	0	0	0	0
MAG092	g__Acinetobacter	s__Acinetobacter johnsonii	95.93	0.66	32	379729	1	1	1	3	0
MAG093	g__UBA1315	s__MAG093	95.88	0.00	66	71069	0	0	0	3	1
MAG094	g__Streptococcus	s__MAG094	95.82	2.70	74	29758	0	0	0	0	0
MAG095	g__KM106-2	s__MAG095	95.72	4.59	267	25327	0	0	0	0	3
MAG096	g__Ligilactobacillus	s__Ligilactobacillus salivarius	95.61	0.52	37	67699	0	1	0	0	0
MAG097	g__Lactococcus	s__Lactococcus lactis	95.60	1.04	1	2076533	1	1	1	18	0

MAGs ID	genus	species	Completeness (%)	Contamination (%)	Num. of scaffolds	N50 of scaffolds (bp)	Num. of 16S rRNA	Num. of 5S rRNA	Num. of 23S rRNA	Num. of rRNA	Genes for Hemi/Cellulose
MAG098	g__Solibacillus	s__MAG098	95.36	1.55	88	70272	0	0	0	0	0
MAG099	g__Abiotrophia	s__MAG099	95.28	1.63	12	403611	2	2	2	3	0
MAG100	g__Proteus	s__Proteus mirabilis	95.22	0.08	345	13592	0	0	0	0	1
MAG101	g__Enterococcus_I	s__MAG101	95.20	2.54	106	38393	0	2	0	1	0
MAG102	g__Paenibacillus_B	s__MAG102	95.19	0.00	158	37339	0	1	0	0	5
MAG103	g__Clostridium	s__MAG103	95.17	0.60	142	35922	0	1	0	0	0
MAG104	g__Ligilactobacillus	s__Ligilactobacillus murinus	95.10	0.00	25	197193	0	1	0	1	0
MAG105	g__Fundicoccus	s__MAG105	95.00	0.45	140	24474	0	0	0	0	2
MAG106	g__Lampropedia	s__MAG106	94.76	0.93	136	39680	0	0	0	0	0
MAG107	g__Streptococcus	s__Streptococcus pasteurianus	94.62	2.81	1	2151585	1	1	1	9	0
MAG108	g__Escherichia	s__Escherichia flexneri	94.61	0.33	30	256635	0	0	0	1	1
MAG109	g__MAG109	s__MAG109	94.58	1.57	108	42901	0	1	0	0	0
MAG110	g__Enterobacter	s__Enterobacter mori	94.54	1.11	44	183499	0	0	0	1	2
MAG111	g__Lactococcus	s__Lactococcus garvieae	94.48	0.38	1	1905862	0	1	0	15	0
MAG112	g__Acinetobacter	s__MAG112	94.48	0.57	29	342642	1	2	0	2	0
MAG113	g__Vagococcus_D	s__MAG113	94.37	3.66	20	577394	3	3	3	0	3
MAG114	g__Chryseobacterium	s__Chryseobacterium piscium	94.32	0.00	180	36017	0	0	0	1	2
MAG115	g__Lactococcus	s__Lactococcus garvieae	94.27	0.00	25	204880	0	0	0	0	0
MAG116	g__Limosilactobacillus	s__Limosilactobacillus ingluvi	94.26	0.00	1	1954091	1	1	1	10	0
MAG117	g__Microbacterium	s__MAG117	94.25	2.82	5	731837	1	1	1	3	1
MAG118	g__Ligilactobacillus	s__MAG118	94.15	6.63	50	51147	0	2	0	0	0
MAG119	g__Bacillus_BN	s__MAG119	94.14	2.17	174	31836	0	1	0	1	0
MAG120	g__YIM-102668	s__MAG120	94.10	2.15	563	15007	0	1	0	0	6
MAG121	g__Enterococcus_G	s__MAG121	94.04	2.65	48	66411	0	0	0	0	1
MAG122	g__Sporosarcina	s__MAG122	94.04	2.21	342	12982	0	0	0	0	1
MAG123	g__Lactococcus	s__Lactococcus garvieae	93.97	0.00	1	1826339	0	1	0	15	0
MAG124	g__Actinomyces	s__MAG124	93.90	2.37	53	106684	2	1	1	0	0
MAG125	g__Conchiformibius	s__MAG125	93.89	0.68	87	33913	0	0	0	0	0
MAG126	g__Enterococcus	s__Enterococcus faecalis	93.52	0.37	1	2436969	1	1	1	6	1
MAG127	g__Streptococcus	s__MAG127	93.46	3.06	99	31036	0	0	0	0	0
MAG128	g__Halomonas	s__Halomonas stevensii	93.46	0.65	62	89654	0	0	0	1	1
MAG129	g__Pseudomonas_E	s__Pseudomonas_E fragi	93.40	8.76	20	256230	0	1	0	0	0
MAG130	g__Lactococcus	s__Lactococcus lactis_E	93.40	0.38	52	118656	0	0	0	0	0

MAGs ID	genus	species	Completeness (%)	Contamination (%)	Num. of scaffolds	N50 of scaffolds (bp)	Num. of 16S rRNA	Num. of 5S rRNA	Num. of 23S rRNA	Num. of rRNA	Genes for Hemi/Cellulose
MAG131	g__MAG131	s__MAG131	93.37	0.38	55	39728	1	0	1	0	0
MAG132	g__Comamonas	s__MAG132	93.36	0.93	83	47570	0	0	0	0	1
MAG133	g__Enterococcus	s__MAG133	93.35	9.88	286	15824	0	0	0	0	1
MAG134	g__Actinomyces	s__MAG134	93.27	2.09	253	14165	0	0	0	0	0
MAG135	g__Comamonas	s__MAG135	93.25	2.10	259	24952	0	0	0	0	0
MAG136	g__Citrobacter	s__Citrobacter portucalensis	93.24	4.50	1	4587216	2	3	2	13	0
MAG137	g__Enterococcus_H	s__Enterococcus_H canis	93.21	1.25	155	24581	0	0	0	0	0
MAG138	g__Oligella	s__Oligella ureolytica	93.21	0.87	218	12914	0	0	0	0	0
MAG139	g__Paenalcaligenes	s__MAG139	93.18	0.53	148	21230	0	0	0	0	0
MAG140	g__Streptococcus	s__Streptococcus orisratti	93.06	7.82	106	38315	0	0	0	1	1
MAG141	g__Clostridium	s__Clostridium neonatale	93.05	0.90	273	20179	0	0	0	0	2
MAG142	g__Bacillus_BN	s__MAG142	93.03	2.50	171	33661	0	0	0	0	0
MAG143	g__Bacillus_BN	s__MAG143	92.99	1.38	22	1712780	5	5	5	6	0
MAG144	g__Streptococcus	s__Streptococcus caballi	92.99	0.37	53	67306	0	0	0	0	2
MAG145	g__Proteus	s__Proteus sp003144405	92.97	5.95	47	131347	0	1	0	0	1
MAG146	g__Enterococcus_I	s__MAG146	92.88	5.85	86	38551	0	0	0	0	0
MAG147	g__Acinetobacter	s__MAG147	92.80	1.83	85	37706	0	1	0	0	0
MAG148	g__Rothia	s__MAG148	92.44	4.75	80	91339	1	2	1	4	0
MAG149	g__Campylobacter_D	s__MAG149	92.41	1.29	64	36535	0	0	0	0	0
MAG150	g__Advenella	s__MAG150	92.38	0.71	296	17189	0	0	0	0	0
MAG151	g__Kluyvera	s__Kluyvera cryocrescens	92.20	1.60	13	645847	1	1	1	12	1
MAG152	g__Jeotgalibaca	s__Jeotgalibaca porci	91.41	2.00	176	13764	0	0	0	1	1
MAG153	g__Chryseobacterium	s__MAG153	91.39	2.94	243	19520	0	0	0	0	2
MAG154	g__Ruminococcus_B	s__Ruminococcus_B gnavus	91.14	1.29	230	14227	0	0	0	0	2
MAG155	g__Limosilactobacillus	s__MAG155	91.09	7.02	53	56992	0	1	0	0	0
MAG156	g__Sphingobacterium	s__MAG156	91.07	4.08	123	63821	0	0	0	0	3
MAG157	g__Flavobacterium	s__MAG157	90.74	5.23	326	11857	0	0	0	0	6
MAG158	g__Comamonas	s__MAG158	90.53	3.24	62	190749	0	0	0	0	0
MAG159	g__Staphylococcus_A	s__Staphylococcus_A lentus	90.52	1.98	264	11561	0	0	0	0	0
MAG160	g__Turicibacter	s__MAG160	90.50	2.12	116	55609	0	0	0	0	0
MAG161	g__Turicibacter	s__MAG161	90.33	2.12	175	29695	0	0	0	0	0
MAG162	g__Raoultella	s__Raoultella electrica	89.73	6.61	45	225313	0	2	0	0	4
MAG163	g__Sphingobacterium	s__Sphingobacterium cavernae	89.63	0.48	282	14723	0	0	0	0	3

MAGs ID	genus	species	Completeness (%)	Contamination (%)	Num. of scaffolds	N50 of scaffolds (bp)	Num. of 16S rRNA	Num. of 5S rRNA	Num. of 23S rRNA	Num. of rRNA	Genes for Hemi/Cellulose
MAG164	g__Limosilactobacillus	s__Limosilactobacillus oris	89.50	0.00	48	61100	0	0	0	0	0
MAG165	g__GCA-900066495	s__GCA-900066495 sp902362	89.46	1.23	355	9750	0	0	0	1	1
MAG166	g__Acinetobacter	s__Acinetobacter sp00302451	89.23	5.48	372	11939	0	0	0	0	0
MAG167	g__Niabella	s__MAG167	89.23	1.24	466	12945	0	0	0	0	9
MAG168	g__Pseudomonas	s__Pseudomonas aeruginosa	89.15	0.60	123	70825	0	1	1	0	0
MAG169	g__Streptococcus	s__Streptococcus ratti	89.12	0.75	72	36933	0	0	0	0	0
MAG170	g__Stenotrophomonas	s__MAG170	89.01	8.26	115	169761	0	0	0	3	0
MAG171	g__Cellulosilyticum	s__MAG171	88.99	5.93	338	17377	0	0	1	2	5
MAG172	g__Streptococcus	s__MAG172	88.79	3.56	1	2041662	1	1	1	11	0
MAG173	g__Streptococcus	s__Streptococcus suis_N	88.47	0.37	29	134162	0	0	0	0	1
MAG174	g__Rahnella	s__Rahnella inusitata	88.40	2.31	46	200282	0	0	0	0	4
MAG175	g__Vagococcus_E	s__MAG175	88.26	0.56	65	65846	0	0	0	0	1
MAG176	g__Ligilactobacillus	s__Ligilactobacillus sp900765	88.22	1.05	37	138583	0	0	0	1	0
MAG177	g__Bergeyella_A	s__MAG177	88.17	0.00	94	30505	0	1	0	0	1
MAG178	g__Pseudomonas_E	s__Pseudomonas_E psychroph	88.10	6.95	1	5739391	2	1	2	15	4
MAG179	g__Streptococcus	s__MAG179	87.99	0.87	175	17275	0	0	0	3	0
MAG180	g__Lysinibacillus	s__MAG180	87.96	0.33	177	30478	1	2	1	6	0
MAG181	g__Acinetobacter	s__MAG181	87.69	1.85	143	25490	0	0	0	3	0
MAG182	g__Paenibacillus	s__MAG182	87.63	1.81	106	92310	3	3	3	10	15
MAG183	g__Oerskovia	s__MAG183	87.31	3.08	39	1157651	2	4	4	0	1
MAG184	g__Streptococcus	s__Streptococcus caballi	86.83	9.68	2	1261907	4	4	4	0	4
MAG185	g__Limosilactobacillus	s__Limosilactobacillus reuteri	86.58	0.00	13	271211	1	1	1	0	0
MAG186	g__Streptococcus	s__Streptococcus porci	86.57	0.39	71	39670	0	0	0	0	1
MAG187	g__Acinetobacter	s__Acinetobacter guillouiae	86.38	3.34	123	50060	0	0	0	2	0
MAG188	g__Leucobacter	s__MAG188	86.36	1.71	349	8521	0	1	0	0	0
MAG189	g__Streptococcus	s__Streptococcus thermophilus	85.96	3.23	144	14845	0	0	0	0	0
MAG190	g__Microlunatus	s__MAG190	85.75	0.69	646	6164	0	0	0	0	6
MAG191	g__Streptococcus	s__Streptococcus sobrinus	85.59	0.55	44	58537	0	0	0	0	0
MAG192	g__Lactococcus	s__Lactococcus petauri	85.59	0.38	13	206583	0	1	0	0	0
MAG193	g__Streptococcus	s__MAG193	85.56	3.20	106	16570	0	0	0	0	0
MAG194	g__Turicibacter	s__MAG194	85.47	3.78	225	14338	0	0	0	0	0
MAG195	g__Cetobacterium_A	s__MAG195	85.44	0.00	79	44862	0	0	0	0	0
MAG196	g__Clostridium	s__MAG196	85.42	9.73	20	394290	6	9	7	3	7

MAGs ID	genus	species	Completeness (%)	Contamination (%)	Num. of scaffolds	N50 of scaffolds (bp)	Num. of 16S rRNA	Num. of 5S rRNA	Num. of 23S rRNA	Num. of rRNA	Genes for Hemi/Cellulose
MAG197	g__Lacticaseibacillus	s__Lacticaseibacillus paracase	85.19	0.00	41	115177	0	1	0	0	0
MAG198	g__Pseudomonas_E	s__Pseudomonas_E fragi	85.04	7.18	15	423117	0	0	0	1	0
MAG199	g__Facklamia_A	s__MAG199	84.86	1.83	262	9061	0	0	0	0	0
MAG200	g__BHER01	s__MAG200	84.72	0.14	24	221544	0	0	0	9	0
MAG201	g__Leuconostoc	s__Leuconostoc citreum	84.72	0.35	1	1485485	1	1	1	9	0
MAG202	g__Stenotrophomonas	s__Stenotrophomonas sp0034{	84.65	5.36	319	16540	0	0	0	3	1
MAG203	g__Escherichia	s__Escherichia flexneri	84.55	3.60	63	105555	0	2	0	0	1
MAG204	g__Acinetobacter	s__Acinetobacter portensis	84.48	5.96	35	184381	1	1	1	9	0
MAG205	g__Cellvibrio	s__MAG205	84.48	1.72	68	139849	0	0	0	0	7
MAG206	g__Lysinibacillus	s__MAG206	84.28	1.32	257	14830	0	0	0	2	0
MAG207	g__Streptococcus	s__Streptococcus pasteurianus	83.90	4.87	14	193971	0	0	0	0	0
MAG208	g__Bacillus_BN	s__MAG208	83.89	2.52	459	8309	0	0	0	1	0
MAG209	g__Comamonas	s__MAG209	83.84	0.93	202	44986	0	0	0	0	0
MAG210	g__Actinomyces	s__Actinomyces slackii	83.78	1.93	260	17275	0	1	0	0	0
MAG211	g__Streptococcus	s__MAG211	83.73	1.25	47	45989	0	0	0	3	0
MAG212	g__Streptococcus	s__MAG212	83.49	3.27	1	1971854	2	2	2	9	0
MAG213	g__Comamonas	s__MAG213	83.43	1.95	512	7755	0	0	0	0	0
MAG214	g__Abiotrophia	s__MAG214	83.37	0.00	118	16519	0	0	0	0	0
MAG215	g__Acinetobacter	s__MAG215	83.30	3.38	194	22158	0	0	0	0	0
MAG216	g__Weissella	s__Weissella confusa_B	83.03	3.07	36	88272	0	3	0	0	0
MAG217	g__Enterococcus_D	s__Enterococcus_D casselifla	83.02	1.28	29	236132	3	3	3	0	1
MAG218	g__Erysipelothrix	s__MAG218	83.02	0.00	177	8746	0	0	0	0	5
MAG219	g__Paucilactobacillus	s__Paucilactobacillus vaccinos	83.02	0.45	74	32849	0	0	0	0	0
MAG220	g__Clostridium_P	s__Clostridium_P mediterrane	82.76	0.00	104	34865	0	1	0	0	0
MAG221	g__Psychrobacter	s__Psychrobacter sanguinis	82.62	0.00	32	85144	0	0	0	0	0
MAG222	g__Dolosicoccus	s__MAG222	82.54	2.13	172	10474	0	0	0	0	0
MAG223	g__Terrisporobacter	s__Terrisporobacter mayombe	82.24	0.00	18	99528	0	0	0	1	0
MAG224	g__Limosilactobacillus	s__MAG224	82.07	0.00	31	107583	0	1	0	1	0
MAG225	g__Clostridium_P	s__Clostridium_P perfringens	81.63	0.56	288	10128	0	0	0	1	0
MAG226	g__Lactiplantibacillus	s__Lactiplantibacillus plantaru	81.48	2.57	29	327951	1	2	1	0	0
MAG227	g__Sphingorhabdus_C	s__MAG227	81.42	3.59	420	7271	0	0	0	0	0
MAG228	g__Paenalcaligenes	s__MAG228	81.42	0.26	196	11517	0	0	0	0	0
MAG229	g__Streptococcus	s__MAG229	81.42	3.85	64	46816	0	0	0	3	0

MAGs ID	genus	species	Completeness (%)	Contamination (%)	Num. of scaffolds	N50 of scaffolds (bp)	Num. of 16S rRNA	Num. of 5S rRNA	Num. of 23S rRNA	Num. of rRNA	Genes for Hemi/Cellulose
MAG230	g__Ligilactobacillus	s__MAG230	80.89	0.52	25	95006	0	1	0	0	0
MAG231	g__Providencia	s__Providencia alcalifaciens	80.69	0.00	1	3253625	2	2	2	3	0
MAG232	g__Streptococcus	s__Streptococcus mitis_BO	80.55	3.78	93	21706	0	0	0	0	0
MAG233	g__Kurthia	s__Kurthia gibsonii	80.42	6.68	356	7997	0	0	0	1	0
MAG234	g__Streptococcus	s__Streptococcus ovis	80.41	4.32	92	27784	0	0	0	0	0
MAG235	g__Helicobacter_A	s__MAG235	80.20	0.47	20	152521	0	0	0	5	0
MAG236	g__Ursidibacter	s__MAG236	80.14	6.47	304	8361	0	0	0	1	0
MAG237	g__Veillonella	s__Veillonella sp900757715	80.13	5.60	168	9880	0	0	0	0	0
MAG238	g__Psychrobacter	s__Psychrobacter sanguinis	79.91	0.76	140	24526	0	0	0	0	0
MAG239	g__Romboutsia_A	s__MAG239	79.66	6.90	555	9072	0	3	0	3	2
MAG240	g__Romboutsia	s__MAG240	79.65	0.35	351	5968	0	0	0	4	1
MAG241	g__Comamonas	s__MAG241	79.55	3.21	26	2699607	3	4	3	3	0
MAG242	g__Aeromonas	s__Aeromonas caviae	79.52	0.58	295	15939	0	0	0	0	1
MAG243	g__Streptococcus	s__Streptococcus sobrinus	79.36	0.94	62	76482	2	2	2	6	0
MAG244	g__Streptococcus	s__Streptococcus caballi	79.28	3.37	36	71136	0	0	0	1	1
MAG245	g__Sphingobacterium	s__MAG245	79.01	5.03	349	16828	0	0	0	0	3
MAG246	g__Corynebacterium	s__Corynebacterium sp001815	78.98	1.37	261	8878	0	0	0	1	0
MAG247	g__Lysinibacillus	s__MAG247	78.04	1.43	376	7812	0	0	0	0	0
MAG248	g__Lactococcus	s__Lactococcus lactis_E	77.81	0.53	149	19238	0	0	0	0	1
MAG249	g__Streptococcus	s__Streptococcus orisratti	77.64	0.20	122	21171	0	0	0	0	2
MAG250	g__Pseudomonas_C	s__Pseudomonas_C sp012719	77.55	0.04	310	7274	0	0	0	0	0
MAG251	g__Acinetobacter	s__MAG251	77.23	4.79	254	17236	1	1	1	0	0
MAG252	g__Pseudomonas_E	s__Pseudomonas_E bubulae	77.19	5.26	89	52092	0	0	0	2	3
MAG253	g__Streptococcus	s__MAG253	76.25	1.17	165	8030	0	0	0	0	0
MAG254	g__Yersinia	s__MAG254	76.21	0.00	45	136906	0	0	0	1	1
MAG255	g__Herminiimonas	s__MAG255	75.98	1.30	369	6751	0	0	0	0	0
MAG256	g__Empedobacter	s__MAG256	75.42	9.87	307	18581	0	0	0	0	6
MAG257	g__Leuconostoc	s__Leuconostoc lactis_A	75.42	9.73	9	1401643	2	2	2	6	0
MAG258	g__Streptococcus	s__Streptococcus halichoeri	75.18	8.94	132	15695	0	0	0	0	0
MAG259	g__Enterococcus_B	s__MAG259	74.45	1.72	171	17466	0	0	0	2	0
MAG260	g__Turicibacter	s__MAG260	73.60	3.28	43	69150	0	0	0	0	0
MAG261	g__Sphingobacterium	s__MAG261	73.54	0.00	69	56514	1	1	1	9	2
MAG262	g__Providencia	s__Providencia alcalifaciens	73.41	1.18	7	2699704	3	3	3	0	0

MAGs ID	genus	species	Completeness (%)	Contamination (%)	Num. of scaffolds	N50 of scaffolds (bp)	Num. of 16S rRNA	Num. of 5S rRNA	Num. of 23S rRNA	Num. of rRNA	Genes for Hemi/Cellulose
MAG263	g__Lelliottia	s__Lelliottia jeotgali	73.11	1.44	6	671239	1	2	1	18	4
MAG264	g__Lysinibacillus	s__MAG264	72.85	1.55	24	157887	0	6	0	1	0
MAG265	g__Neisseria	s__MAG265	72.84	1.97	241	8603	0	0	0	0	0
MAG266	g__Streptococcus	s__Streptococcus caballi	72.58	7.38	52	72790	0	0	0	0	0
MAG267	g__Lactobacillus	s__Lactobacillus johnsonii	72.41	9.48	244	8102	0	0	0	1	0
MAG268	g__Clostridium	s__Clostridium thermobutyricu	72.41	0.00	7	2435311	4	6	4	4	0
MAG269	g__Clostridium_P	s__MAG269	72.41	0.00	24	126939	0	1	0	1	0
MAG270	g__Atopostipes	s__MAG270	72.25	1.69	233	7013	0	0	0	0	1
MAG271	g__Acinetobacter	s__MAG271	72.05	0.00	318	11383	0	0	0	0	0
MAG272	g__Acinetobacter	s__Acinetobacter johnsonii	71.98	2.17	483	5068	0	0	0	0	0
MAG273	g__Streptococcus	s__MAG273	71.46	0.78	151	11982	0	1	0	0	0
MAG274	g__Pantoea	s__Pantoea ananatis	71.19	3.45	313	20872	0	0	0	0	4
MAG275	g__Acinetobacter	s__MAG275	71.19	1.72	336	10264	0	0	0	0	0
MAG276	g__Plesiomonas	s__Plesiomonas shigelloides	70.90	1.13	14	210134	5	5	5	16	1
MAG277	g__Enterococcus_A	s__Enterococcus_A avium	70.79	4.85	371	11545	0	0	0	0	0
MAG278	g__Acinetobacter	s__Acinetobacter sp00236745	70.69	9.48	75	43770	0	0	0	0	0
MAG279	g__Vagococcus_D	s__MAG279	70.69	6.03	92	416804	2	3	2	0	1
MAG280	g__Sporosarcina	s__Sporosarcina ureae_C	70.69	1.72	244	16324	0	0	0	1	2
MAG281	g__Terrisporobacter	s__Terrisporobacter mayombe	70.69	1.72	42	92372	0	0	0	9	2
MAG282	g__Acinetobacter	s__Acinetobacter towneri	70.64	2.59	172	25012	0	0	0	0	0
MAG283	g__Serratia	s__Serratia ureilytica	70.52	0.00	1	3299453	3	4	3	9	1
MAG284	g__Streptococcus	s__Streptococcus ferus	70.45	0.00	95	19193	0	0	0	0	0
MAG285	g__Klebsiella	s__Klebsiella pneumoniae	70.23	5.01	18	645200	0	0	0	16	3
MAG286	g__Atopostipes	s__MAG286	70.14	0.00	195	6969	0	0	0	1	0
MAG287	g__Clostridium	s__Clostridium butyricum	70.13	5.17	573	6748	0	1	0	0	0
MAG288	g__Acinetobacter	s__Acinetobacter gandensis	70.07	3.66	37	108608	0	0	0	0	0
MAG289	g__MAG289	s__MAG289	70.03	1.94	436	5168	0	0	0	0	4
MAG290	g__Jeotgalicoccus	s__MAG290	69.40	1.34	230	6822	0	0	0	0	0
MAG291	g__Abiotrophia	s__MAG291	69.34	1.90	59	32064	0	0	0	0	0
MAG292	g__Pseudomonas_C	s__MAG292	69.31	1.72	257	10502	0	0	0	0	0
MAG293	g__Lactococcus	s__Lactococcus lactis	69.14	4.45	1	1687929	4	4	4	1	0
MAG294	g__Clostridium	s__Clostridium paraputrificum	69.06	1.56	383	5408	0	1	0	1	0
MAG295	g__Pediococcus	s__Pediococcus pentosaceus	68.97	0.00	3	406260	0	0	0	0	0

MAGs ID	genus	species	Completeness (%)	Contamination (%)	Num. of scaffolds	N50 of scaffolds (bp)	Num. of 16S rRNA	Num. of 5S rRNA	Num. of 23S rRNA	Num. of rRNA	Genes for Hemi/Cellulose
MAG296	g__Chryseobacterium	s__MAG296	68.83	6.03	273	8097	0	1	0	0	0
MAG297	g__Chryseobacterium	s__Chryseobacterium sp00129	68.76	3.45	492	15501	0	0	0	0	1
MAG298	g__Helicobacter_A	s__MAG298	68.19	0.12	77	26695	0	0	0	0	0
MAG299	g__Weissella	s__Weissella cibaria	67.89	0.00	219	10425	0	0	0	0	0
MAG300	g__Enterococcus_I	s__Enterococcus_I aquimarinu	67.63	0.22	194	7095	0	2	0	0	0
MAG301	g__Microlunatus	s__MAG301	67.32	2.07	526	6701	0	0	0	0	3
MAG302	g__Stenotrophomonas	s__MAG302	67.24	1.72	210	22829	0	0	0	0	0
MAG303	g__Cetobacterium_A	s__Cetobacterium_A somerae	67.24	0.00	8	342115	0	1	0	0	0
MAG304	g__Helicobacter_A	s__Helicobacter_A muridarum	67.03	0.77	253	5682	0	0	0	0	0
MAG305	g__VLXZ01	s__MAG305	67.00	5.50	436	6127	0	1	0	1	1
MAG306	g__Citrobacter	s__Citrobacter murliniae	66.82	0.85	27	225640	0	2	0	3	0
MAG307	g__Ursidibacter	s__MAG307	66.81	0.80	258	6874	0	0	0	0	0
MAG308	g__Jeotgalicoccus	s__Jeotgalicoccus nanhaiensis	66.70	0.77	216	7796	0	0	0	0	0
MAG309	g__Enterococcus_C	s__Enterococcus_C sp0099331	66.70	1.72	365	6221	0	0	0	0	1
MAG310	g__Sphingobacterium	s__MAG310	66.55	0.00	262	13035	0	0	0	0	1
MAG311	g__Lactococcus	s__Lactococcus lactis	66.38	7.48	8	484350	2	2	2	4	0
MAG312	g__Leuconostoc	s__Leuconostoc lactis	66.22	9.72	30	158003	0	0	0	0	0
MAG313	g__Empedobacter	s__MAG313	65.59	0.00	99	20264	0	0	0	0	1
MAG314	g__Klebsiella	s__Klebsiella pneumoniae	65.52	5.17	24	360915	2	2	2	3	0
MAG315	g__Lelliottia	s__MAG315	65.52	0.00	3	3891227	3	3	3	12	6
MAG316	g__Bacillus_BN	s__MAG316	65.52	0.00	1	2276866	1	1	1	1	0
MAG317	g__Lactobacillus	s__Lactobacillus kitasatonis	64.61	0.00	8	348923	0	0	0	0	0
MAG318	g__Providencia	s__MAG318	64.17	4.49	7	659577	3	4	3	6	0
MAG319	g__MAG319	s__MAG319	63.96	1.91	83	49854	3	3	3	3	1
MAG320	g__Enterobacter	s__Enterobacter hormaechei_A	63.95	5.17	229	32623	0	0	0	2	1
MAG321	g__Clostridium	s__MAG321	63.79	1.72	121	24570	0	0	0	0	0
MAG322	g__Acinetobacter	s__Acinetobacter baumannii	63.79	0.00	164	20400	0	0	0	0	0
MAG323	g__Kosakonia	s__Kosakonia cowanii	63.75	5.17	75	133227	0	2	0	3	2
MAG324	g__Paeniglutamicibacter	s__MAG324	63.40	0.86	556	8846	0	0	0	0	0
MAG325	g__Acinetobacter	s__MAG325	63.28	1.72	263	20354	0	0	0	0	0
MAG326	g__Pseudomonas_E	s__Pseudomonas_E fragi	63.16	0.00	32	412599	1	1	1	3	1
MAG327	g__Clostridium	s__MAG327	63.04	2.53	125	24895	0	0	0	0	1
MAG328	g__Enterococcus_B	s__Enterococcus_B faecium	62.92	0.00	33	82242	0	0	0	0	0

MAGs ID	genus	species	Completeness (%)	Contamination (%)	Num. of scaffolds	N50 of scaffolds (bp)	Num. of 16S rRNA	Num. of 5S rRNA	Num. of 23S rRNA	Num. of rRNA	Genes for Hemi/Cellulose
MAG329	g__Timonella	s__MAG329	62.35	0.00	236	7535	0	0	0	0	0
MAG330	g__Pseudomonas_E	s__MAG330	62.24	2.07	689	7072	0	0	0	0	0
MAG331	g__Lelliottia	s__MAG331	62.07	5.17	177	33903	0	0	0	0	0
MAG332	g__Acinetobacter	s__Acinetobacter albensis	62.07	0.00	320	9045	0	1	0	1	0
MAG333	g__Sphingobacterium	s__Sphingobacterium nematoc	61.55	0.00	333	10107	0	0	0	0	2
MAG334	g__Lactobacillus	s__MAG334	61.21	0.00	95	30058	0	0	0	3	0
MAG335	g__Vagococcus_D	s__MAG335	61.18	4.25	576	11521	0	0	0	0	5
MAG336	g__Klebsiella	s__Klebsiella variicola	60.85	0.00	62	154041	0	0	0	0	2
MAG337	g__Acinetobacter	s__MAG337	60.83	8.09	47	186479	0	0	0	3	0
MAG338	g__Buttiauxella	s__MAG338	60.69	1.72	84	139276	0	0	0	0	2
MAG339	g__Enterococcus_A	s__Enterococcus_A avium	60.69	2.31	112	51137	2	2	2	2	0
MAG340	g__Lactococcus	s__Lactococcus lactis	60.67	7.20	44	63093	1	2	1	12	0
MAG341	g__Weissella	s__Weissella confusa	60.53	0.00	152	16558	0	1	0	0	0
MAG342	g__Lactobacillus	s__Lactobacillus acidophilus	60.37	3.04	22	91293	1	1	1	6	0
MAG343	g__Paenibacillus	s__MAG343	60.34	1.72	240	27411	0	0	0	3	7
MAG344	g__Escherichia	s__Escherichia dysenteriae	60.34	1.72	44	86088	0	0	0	1	2
MAG345	g__MAG345	s__MAG345	60.31	1.00	250	5088	0	1	0	0	0
MAG346	g__Bacillus_BN	s__MAG346	60.18	7.74	14	474957	1	4	2	10	0
MAG347	g__Comamonas	s__MAG347	60.13	0.36	7	403011	0	0	0	0	0
MAG348	g__Hafnia	s__Hafnia alvei	60.09	1.78	78	51258	0	1	0	0	2
MAG349	g__Lysinibacillus	s__MAG349	59.65	7.02	82	50681	0	0	0	1	0
MAG350	g__Cellulosilyticum	s__MAG350	59.65	0.00	45	47445	0	0	0	0	0
MAG351	g__Pseudomonas_E	s__MAG351	59.62	4.18	23	142107	0	1	0	3	0
MAG352	g__Oerskovia	s__MAG352	59.55	5.88	1708	4039	0	0	0	0	0
MAG353	g__Paracoccus	s__MAG353	59.25	3.52	592	4690	0	0	0	0	0
MAG354	g__Enterococcus_B	s__Enterococcus_B pernyi	58.62	6.90	9	176196	0	0	0	0	0
MAG355	g__Turicibacter	s__MAG355	58.62	0.00	313	10719	0	0	0	0	0
MAG356	g__Campylobacter_D	s__MAG356	58.62	0.00	55	33628	0	0	0	0	0
MAG357	g__Vagococcus_D	s__Vagococcus_D salmoninarı	58.22	0.86	20	340862	5	6	5	0	0
MAG358	g__Cellulosilyticum	s__MAG358	57.89	3.51	32	69576	0	0	0	0	2
MAG359	g__Limosilactobacillus	s__Limosilactobacillus reuteri	57.44	2.30	20	125380	0	0	0	0	0
MAG360	g__YIM-102668	s__MAG360	57.35	0.00	327	4341	0	0	0	0	1
MAG361	g__Acinetobacter	s__MAG361	57.35	0.00	126	18224	0	0	0	0	0

MAGs ID	genus	species	Completeness (%)	Contamination (%)	Num. of scaffolds	N50 of scaffolds (bp)	Num. of 16S rRNA	Num. of 5S rRNA	Num. of 23S rRNA	Num. of rRNA	Genes for Hemi/Cellulose
MAG362	g__Terrisporobacter	s__MAG362	57.24	0.00	541	6874	0	0	0	3	0
MAG363	g__Pseudomonas_E	s__MAG363	57.02	0.00	215	31711	0	0	0	0	2
MAG364	g__Comamonas	s__MAG364	56.71	9.76	193	35944	0	0	0	0	0
MAG365	g__MAG365	s__MAG365	56.44	0.98	288	7590	0	0	0	0	0
MAG366	g__Chryseobacterium	s__MAG366	56.27	4.00	313	10108	0	0	0	0	0
MAG367	g__Necropsobacter	s__Necropsobacter rosorum	56.26	0.69	122	16160	0	0	0	0	0
MAG368	g__Chryseobacterium	s__MAG368	56.23	0.98	74	62092	0	0	0	0	0
MAG369	g__Citrobacter	s__Citrobacter portucalensis	56.19	1.72	66	108595	0	1	0	1	0
MAG370	g__Acinetobacter	s__MAG370	56.03	2.59	92	52934	0	0	0	5	0
MAG371	g__Basfia_A	s__MAG371	56.03	0.56	5	771672	4	6	4	0	0
MAG372	g__Conchiformibius	s__MAG372	56.01	0.79	69	27774	1	1	1	3	0
MAG373	g__Carnobacterium	s__Carnobacterium maltaroma	55.95	1.73	38	80493	1	1	1	0	2
MAG374	g__Hafnia	s__Hafnia proteus	55.64	1.72	177	41335	3	3	3	4	3
MAG375	g__Klebsiella_A	s__Klebsiella_A oxytoca	55.42	8.77	194	43797	0	0	0	0	1
MAG376	g__Pseudomonas_E	s__Pseudomonas_E parafulva_	54.74	0.00	542	8280	0	1	0	0	0
MAG377	g__Glutamicibacter	s__Glutamicibacter sp0029798	54.47	0.00	335	13520	0	0	0	0	2
MAG378	g__Pseudomonas_E	s__Pseudomonas_E lundensis	54.39	1.75	154	24814	0	0	0	0	0
MAG379	g__Acinetobacter	s__Acinetobacter baumannii	54.31	6.90	217	13952	0	0	0	0	0
MAG380	g__Acinetobacter	s__MAG380	54.31	1.72	128	26958	1	1	1	0	0
MAG381	g__Arthrobacter_A	s__MAG381	54.31	0.00	478	5096	0	0	0	0	0
MAG382	g__MAG382	s__MAG382	54.30	0.88	103	27123	0	0	0	3	0
MAG383	g__Serratia	s__Serratia marcescens_I	53.95	1.72	28	198171	0	0	0	1	1
MAG384	g__Erwinia	s__Erwinia sp001422605	53.61	0.00	489	10830	0	0	0	0	2
MAG385	g__Pantoea	s__MAG385	53.61	0.00	696	4467	0	0	0	0	1
MAG386	g__Stenotrophomonas	s__Stenotrophomonas sp0030[illegible]	53.45	6.90	700	3875	0	0	0	0	2
MAG387	g__Sphingobacterium	s__MAG387	53.45	5.17	416	9289	0	0	0	0	1
MAG388	g__Acinetobacter	s__Acinetobacter sp00371139[illegible]	53.45	1.72	322	8464	0	0	0	0	0
MAG389	g__Clostridium	s__MAG389	53.45	1.72	14	242122	0	0	0	0	1
MAG390	g__Escherichia	s__Escherichia coli	53.45	0.00	15	572323	0	0	0	0	1
MAG391	g__Acinetobacter	s__MAG391	53.32	2.89	76	63739	0	0	0	3	0
MAG392	g__MAG392	s__MAG392	53.16	1.27	20	117292	0	0	0	0	0
MAG393	g__Pseudomonas_E	s__Pseudomonas_E qingdaone	52.63	1.75	62	63567	0	1	0	0	1
MAG394	g__Weissella	s__Weissella cibaria	52.63	0.00	64	46090	0	0	0	0	0

MAGs ID	genus	species	Completeness (%)	Contamination (%)	Num. of scaffolds	N50 of scaffolds (bp)	Num. of 16S rRNA	Num. of 5S rRNA	Num. of 23S rRNA	Num. of rRNA	Genes for Hemi/Cellulose
MAG395	g__Enterococcus_B	s__MAG395	52.07	6.90	550	5122	0	0	0	0	5
MAG396	g__Arthrobacter_A	s__MAG396	51.75	1.72	445	8282	0	0	0	0	1
MAG397	g__Pseudomonas_C	s__MAG397	51.72	0.86	334	6909	0	0	0	0	1
MAG398	g__Staphylococcus_A	s__Staphylococcus_A pasteuri	51.72	0.00	314	6310	0	0	0	0	0
MAG399	g__Streptococcus	s__Streptococcus orisratti	51.72	0.00	2	953386	1	1	1	0	1
MAG400	g__Comamonas	s__MAG400	51.10	0.93	74	95421	0	0	0	0	0
MAG401	g__Raoultella	s__Raoultella ornithinolytica	50.88	8.77	206	41508	0	0	0	0	4
MAG402	g__Escherichia	s__Escherichia marmotae	50.86	1.72	81	172898	0	1	0	0	1
MAG403	g__Abiotrophia	s__MAG403	50.78	0.00	69	22230	0	0	0	0	0
MAG404	g__Kluyvera	s__Kluyvera cryocrescens	50.43	0.49	2	1554579	5	6	4	4	2
MAG405	g__Paenalcaligenes	s__Paenalcaligenes sp0128395	50.17	1.72	211	5936	0	0	0	0	0
MAG406	g__Klebsiella	s__Klebsiella quasipneumonia	50.00	3.45	83	247392	0	0	0	6	2
MAG407	g__Lactococcus	s__Lactococcus lactis_E	50.00	3.30	22	112343	0	1	0	0	1
MAG408	g__Klebsiella	s__Klebsiella aerogenes	50.00	0.00	28	205175	0	0	0	1	1

MAGs ID	genus	species	Completeness (%)	Contamination (%)	Num. of scaffolds	N50 of scaffolds (bp)	Num. of 16S rRNA	Num. of 5S rRNA	Num. of 23S rRNA	Num. of rRNA
MAG389	g__Clostridium	s__MAG389	53.448	1.724	14	242122	0	0	0	0
MAG390	g__Escherichia	s__Escherichia coli	53.448	0.000	15	572323	0	0	0	0
MAG391	g__Acinetobacter	s__MAG391	53.321	2.893	76	63739	0	0	0	3
MAG392	g__MAG392	s__MAG392	53.165	1.266	20	117292	0	0	0	0
MAG393	g__Pseudomonas_E	s__Pseudomonas_E qingdaonensis	52.632	1.754	62	63567	0	1	0	0
MAG394	g__Weissella	s__Weissella cibaria	52.632	0.000	64	46090	0	0	0	0
MAG395	g__Enterococcus_B	s__MAG395	52.069	6.897	550	5122	0	0	0	0
MAG396	g__Arthrobacter_A	s__MAG396	51.750	1.724	445	8282	0	0	0	0
MAG397	g__Pseudomonas_C	s__MAG397	51.724	0.862	334	6909	0	0	0	0
MAG398	g__Staphylococcus_A	s__Staphylococcus_A pasteuri	51.724	0.000	314	6310	0	0	0	0
MAG399	g__Streptococcus	s__Streptococcus orisratti	51.724	0.000	2	953386	1	1	1	0
MAG400	g__Comamonas	s__MAG400	51.096	0.929	74	95421	0	0	0	0
MAG401	g__Raoultella	s__Raoultella ornithinolytica	50.877	8.772	206	41508	0	0	0	0
MAG402	g__Escherichia	s__Escherichia marmotae	50.862	1.724	81	172898	0	1	0	0
MAG403	g__Abiotrophia	s__MAG403	50.784	0.000	69	22230	0	0	0	0
MAG404	g__Kluyvera	s__Kluyvera cryocrescens	50.430	0.489	2	1554579	5	6	4	4
MAG405	g__Paenalcaligenes	s__Paenalcaligenes sp012839585	50.172	1.724	211	5936	0	0	0	0
MAG406	g__Klebsiella	s__Klebsiella quasipneumoniae	50.000	3.448	83	247392	0	0	0	6
MAG407	g__Lactococcus	s__Lactococcus lactis_E	50.000	3.302	22	112343	0	1	0	0
MAG408	g__Klebsiella	s__Klebsiella aerogenes	50.000	0.000	28	205175	0	0	0	1

Table S3. Metatranscriptomic sequencing profile of selected samples

SampleID	# of RawReads	# of clean reads (paired)	# of nonRNA reads	Reads length	RawData (bp)	nonRNA bases (bp)
DD10T	30968938	28202412	27253403	150	9290681400	8176020900
DD2T	35744545	33927330	33453227	150	10723363500	10035968100
DD4T	36071935	33375153	32515007	150	10821580500	9754502100
DD8T	35830128	30125253	29293653	150	10749038400	8788095900
DG1T	36382723	34406187	33533473	150	10914816900	10060041900
DG3T	39351470	37287982	36518295	150	11805441000	10955488500
DG4T	40981324	35587300	34217993	150	12294397200	10265397900
DG5T	38538014	36329171	35210671	150	11561404200	10563201300
DJ1T	32835620	29894686	29192954	150	9850686000	8757886200
DJ2T	33100432	30854042	30108718	150	9930129600	9032615400
DJ4T	34926831	32847326	31880960	150	10478049300	9564288000
DJ5T	31466718	26648618	26138023	150	9440015400	7841406900
DJ6T	41643000	39051613	37594357	150	12492900000	11278307100
DJ7T	45256504	42858372	41934164	150	13576951200	12580249200

Table S4 Selected enzymes linked with cellulose degration, amino acide degradation, and amino acid biosynthesis

Cellulose degradation related ENZYME entry	Amino acid degradation related ENZYME entry		Amino acid byosynthesis related ENZYME entry	
EC 1.1.3.7	EC 1.1.1.157	EC 4.1.3.39	EC 1.1.1.23	EC 2.6.1.78
EC 1.10.3.2	EC 1.1.1.35	EC 4.1.3.4	EC 1.1.1.3	EC 2.6.1.79
EC 1.11.1.13	EC 1.13.11.16	EC 4.2.1.17	EC 1.1.1.85	EC 2.6.1.83
EC 1.11.1.14	EC 1.13.11.27	EC 4.2.1.18	EC 1.1.1.86	EC 2.6.1.9
EC 1.13.11.2	EC 1.13.11.5	EC 4.2.1.22	EC 1.1.1.87	EC 2.7.1.39
EC 1.14.99.54	EC 1.14.12.19	EC 4.2.1.49	EC 1.1.1.95	EC 2.7.2.11
EC 1.14.99.56	EC 1.14.13.127	EC 4.2.1.80	EC 1.17.1.8	EC 2.7.2.124
EC 1.6.5.5	EC 1.14.13.149	EC 4.3.1.3	EC 1.2.1.103	EC 2.7.2.17
EC 2.4.1.20	EC 1.2.1.10	EC 5.2.1.2	EC 1.2.1.106	EC 2.7.2.19
EC 3.1.1.3	EC 1.2.1.16	EC 5.3.3.18	EC 1.2.1.11	EC 2.7.2.4
EC 3.1.1.72	EC 1.2.1.24	EC 6.2.1.30	EC 1.2.1.38	EC 2.7.2.8
EC 3.1.1.73	EC 1.2.1.31	EC 6.4.1.4	EC 1.2.1.41	EC 3.1.3.15
EC 3.1.6.7	EC 1.2.1.79		EC 1.2.1.95	EC 3.1.3.3
EC 3.2.1.139	EC 1.2.1.91		EC 1.3.1.12	EC 3.5.1.130
EC 3.2.1.176	EC 1.2.4.4		EC 1.3.1.13	EC 3.5.1.132
EC 3.2.1.203	EC 1.3.1.87		EC 1.3.1.43	EC 3.5.1.14
EC 3.2.1.21	EC 1.3.8.4		EC 1.3.1.78	EC 3.5.1.16
EC 3.2.1.37	EC 1.3.8.6		EC 1.4.1.16	EC 3.5.1.18
EC 3.2.1.4	EC 1.3.8.7		EC 1.5.1.10	EC 3.5.1.47
EC 3.2.1.55	EC 1.4.1.1		EC 1.5.1.2	EC 3.5.4.19
EC 3.2.1.72	EC 1.4.1.3		EC 1.5.1.7	EC 3.6.1.31
EC 3.2.1.73	EC 1.4.1.5		EC 2.1.1.13	EC 4.1.1.20
EC 3.2.1.74	EC 1.4.3.2		EC 2.1.1.14	EC 4.1.1.48
EC 3.2.1.8	EC 1.5.1.10		EC 2.1.3.3	EC 4.1.3.27
EC 3.2.1.86	EC 1.5.1.7		EC 2.1.3.9	EC 4.2.1.108
EC 3.2.1.91	EC 1.5.1.8		EC 2.2.1.6	EC 4.2.1.114
EC 3.2.1.99	EC 1.5.1.9		EC 2.3.1.1	EC 4.2.1.19
	EC 1.8.1.4		EC 2.3.1.117	EC 4.2.1.20
	EC 2.1.1.37		EC 2.3.1.178	EC 4.2.1.22
	EC 2.1.2.5		EC 2.3.1.30	EC 4.2.1.33
	EC 2.3.1.168		EC 2.3.1.35	EC 4.2.1.35
	EC 2.3.1.174		EC 2.3.1.46	EC 4.2.1.36
	EC 2.3.1.233		EC 2.3.1.89	EC 4.2.1.51
	EC 2.5.1.6		EC 2.3.3.13	EC 4.2.1.9
	EC 2.6.1.19		EC 2.3.3.14	EC 4.2.1.91
	EC 2.6.1.39		EC 2.3.3.21	EC 4.2.3.1
	EC 2.6.1.42		EC 2.4.2.17	EC 4.3.1.19
	EC 2.6.1.5		EC 2.4.2.18	EC 4.3.2.1
	EC 2.6.1.57		EC 2.5.1.134	EC 4.3.2.10
	EC 2.6.1.96		EC 2.5.1.47	EC 4.3.3.7
	EC 3.3.1.1		EC 2.5.1.48	EC 4.4.1.1
	EC 3.3.2.12		EC 2.6.1.11	EC 4.4.1.13
	EC 3.5.1.68		EC 2.6.1.118	EC 5.1.1.7
	EC 3.5.2.7		EC 2.6.1.17	EC 5.3.1.16
	EC 3.5.3.13		EC 2.6.1.39	EC 5.3.1.24
	EC 3.5.3.8		EC 2.6.1.42	EC 5.4.99.5
	EC 3.7.1.14		EC 2.6.1.52	EC 6.3.2.43
	EC 3.7.1.2		EC 2.6.1.57	EC 6.3.2.60
	EC 4.1.1.15		EC 2.6.1.76	EC 6.3.4.5

Table S5. Comparison of amino acid abundance among different treatment group in mice

	Name	Mean_LC	Dev_LC	Mean_LS	Dev_LS	T-test	Mean_NC	Dev_NC	Mean_NS	Dev_NS	T-test
Non-essential amino-acid	L-Tyrosine	6.17E+10	6.80E+09	7.06E+10	1.04E+10	0.026853	5.89E+10	1.23E+10	6.27E+10	9.21E+09	0.397279
	L-Histidine	3.90E+09	2.11E+09	5.62E+09	1.99E+09	0.062944	3.16E+09	1.82E+09	3.83E+09	2.46E+09	0.457338
	L-Cysteine	2.96E+08	1.66E+08	1.77E+08	1.37E+08	0.081602	2.46E+08	1.46E+08	2.35E+08	1.59E+08	0.860349
	L-Glutamic acid	1.37E+10	3.26E+09	1.54E+10	4.18E+09	0.309275	1.17E+10	2.06E+09	1.42E+10	3.14E+09	0.032542
	L-Asparagine	1.34E+07	6.65E+06	1.60E+07	7.36E+06	0.395013	9.60E+06	1.33E+06	1.02E+07	1.97E+06	0.372636
	L-Arginine	2.95E+06	7.94E+05	3.50E+06	1.97E+06	0.405963	2.76E+06	1.04E+06	2.67E+06	9.24E+05	0.823397
	L-Serine	1.42E+09	4.95E+08	1.53E+09	4.80E+08	0.584206	1.16E+09	1.99E+08	1.46E+09	4.95E+08	0.062174
	L-Aspartic acid	2.45E+09	8.92E+08	2.66E+09	9.58E+08	0.611999	2.06E+09	3.08E+08	2.52E+09	8.49E+08	0.089027
	L-Ornithine	3.89E+07	4.14E+07	3.80E+07	5.06E+07	0.961997	1.50E+07	4.47E+06	2.31E+07	1.65E+07	0.114386
	NEAA Total	8.35E+10	7.90E+09	9.61E+10	1.51E+10	0.023677	7.72E+10	1.53E+10	8.50E+10	1.34E+10	0.200999
Essential amino-acid	L-Lysine	1.51E+06	4.55E+05	1.50E+06	6.66E+05	0.97829	1.43E+06	6.08E+05	1.50E+06	5.93E+05	0.766017
	L-Valine	2.85E+10	5.33E+09	3.83E+10	1.37E+10	0.039086	2.63E+10	7.25E+09	3.12E+10	6.91E+09	0.101319
	L-Threonine	5.30E+09	8.08E+08	5.86E+09	1.25E+09	0.22531	4.81E+09	7.92E+08	5.58E+09	1.12E+09	0.065288
	L-Phenylalanine	3.21E+09	1.48E+09	4.10E+09	1.85E+09	0.227666	2.61E+09	9.96E+08	3.04E+09	1.26E+09	0.366806
	L-Tryptophan	4.13E+06	2.01E+06	4.49E+06	2.05E+06	0.683681	3.40E+06	1.13E+06	4.60E+06	3.55E+06	0.276671
	EAA Total	3.71E+10	6.53E+09	4.83E+10	1.58E+10	0.041721	3.37E+10	8.65E+09	3.98E+10	8.18E+09	0.087694
